# The NUDIX hydrolase NUDT5 regulates thiopurine metabolism and cytotoxicity

**DOI:** 10.1172/JCI190443

**Published:** 2025-07-15

**Authors:** Maud Maillard, Rina Nishii, Hieu S. Vu, Kashi R. Bhattarai, Wenjian Yang, Jing Li, Ute Hofmann, Daniel Savic, Smita Bhatia, Matthias Schwab, Min Ni, Jun J. Yang

**Affiliations:** 1 Department of Pharmacy and Pharmaceutical Sciences and; 2 Department of Pathology, Center of Excellence for Leukemia Studies, St. Jude Children’s Research Hospital, Memphis, Tennessee, USA.; 3 Karmanos Cancer Institute, Wayne State University School of Medicine, Detroit, Michigan, USA.; 4 Dr. Margarete Fischer-Bosch Institute of Clinical Pharmacology, Stuttgart, Germany.; 5 Institute for Cancer Outcomes and Survivorship and Division of Pediatric Hematology-Oncology, University of Alabama at Birmingham, Birmingham, Alabama, USA.; 6 Departments of Clinical Pharmacology, and of Biochemistry and Pharmacy and; 7 Cluster of Excellence iFIT (EXC 2180) “Image-guided and Functionally Instructed Tumor Therapies,” University Tuebingen, Tuebingen, Germany.; 8 Department of Oncology, St. Jude Children’s Research Hospital, Memphis, Tennessee, USA.

**Keywords:** Genetics, Oncology, Therapeutics, Pharmacogenetics

## Abstract

Thiopurines are anticancer agents used for the treatment of leukemia and autoimmune diseases. These purine analogs are characterized by a narrow therapeutic index because of the risk of myelosuppression. With the discovery of NUDIX hydrolase 15 (NUDT15) as a major modulator of thiopurine metabolism and toxicity, we sought to comprehensively examine all members of the NUDIX hydrolase family for their effect on the pharmacologic effects of thiopurine. By performing a *NUDIX*-targeted CRISPR/Cas9 screen in leukemia cells, we identified *NUDT5*, whose depletion led to drastic thiopurine resistance. NUDT5 deficiency resulted in a nearly complete depletion of active metabolites of thiopurine and the loss of thioguanine incorporation into DNA. Mechanistically, NUDT5 deletion resulted in substantial alteration in purine nucleotide biosynthesis, as determined by steady-state metabolomics profiling. Stable isotope tracing demonstrated that the loss of *NUDT5* was linked to a marked suppression of the purine salvage pathway but with minimal effects on purine de novo synthesis. Finally, we comprehensively identified germline genetic variants in *NUDT5* associated with thiopurine-induced myelosuppression in 582 children with acute lymphoblastic leukemia. Collectively, these results pointed to NUDT5 as a key regulator of the thiopurine response primarily through its effects on purine homeostasis, highlighting its potential to inform individualized thiopurine therapy.

## Abstract 2

NUDT5 is a key regulator of thiopurine response through its effects on purine homeostasis and has a potential to inform individualized thiopurine therapy.

## Introduction

Thiopurines, including 6-thioguanine (TG), 6-mercaptopurine (MP), and its prodrug azathioprine, are widely used therapeutic agents for treating cancers such as acute lymphoblastic leukemia (ALL), as well as autoimmune diseases like inflammatory bowel disease (IBD) (1, 2). However, these synthetic guanine analogs have a narrow therapeutic index due to severe hematopoietic toxicity that can lead to treatment discontinuation and life-threatening infections (3, 4).

Thiopurines exert their antileukemic activity only after being extensively metabolized into thioguanine nucleotides (TGNs). MP and TG are first converted by hypoxanthine-guanine phosphoribosyl transferase (HPRT) into thioinosine monophosphate (TIMP) and thioguanosine monophosphate (TGMP), respectively, and subsequently into active cytotoxic thioguanosine triphosphate (TGTP). The incorporation of TGTP into DNA (as DNA-TG) triggers post-replication mismatch repair (MMR), DNA strand breaks, and, ultimately, apoptosis (5–7). Thiopurine methyltransferase (TPMT) and NUDIX hydrolase 15 (NUDT15) are 2 major metabolizing enzymes of thiopurines, both acting to prevent the excessive build-up of TGNs. More important, germline loss-of-function variants in these 2 genes have been associated with thiopurine toxicity (8–18). This led to the recommendations of a *TPMT*/*NUDT15* genotype–guided dose adjustment by the Clinical Pharmacogenetics Implementation Consortium (CPIC) (19), which have since been added to the drug labels issued by the US FDA, the European Medicines Agency, and other regulatory agencies globally.

NUDT15 belongs to a family of 22 hydrolases that dephosphorylate a wide variety of naturally occurring nucleotides as well as synthetic analogs that are used in cancer or infectious disease therapies (20, 21). With the exception of NUDT15, the role of these enzymes in thiopurine disposition has not been systematically examined. Yet, the promiscuity in their substrate recognition suggests that other NUDIX enzymes may also be involved in regulating thiopurine metabolism and/or response.

In this study, we performed a *NUDIX*-targeted CRISPR/Cas9 screen in human B-ALL cells treated with thiopurine and found that NUDIX hydrolase 5–deficient cells were resistant to thiopurine. We performed extensive metabolomics profiling followed by stable isotope tracing of the purine synthesis pathway to identify the molecular mechanisms supporting this phenotype. Finally, we systematically evaluated the association of germline genetic variants in *NUDT5* with thiopurine-related myelosuppression in 582 children with ALL.

## Results

### NUDIX-targeted CRISPR/Cas9 screen for modulators of thiopurine cytotoxicity.

**Figure 1:**
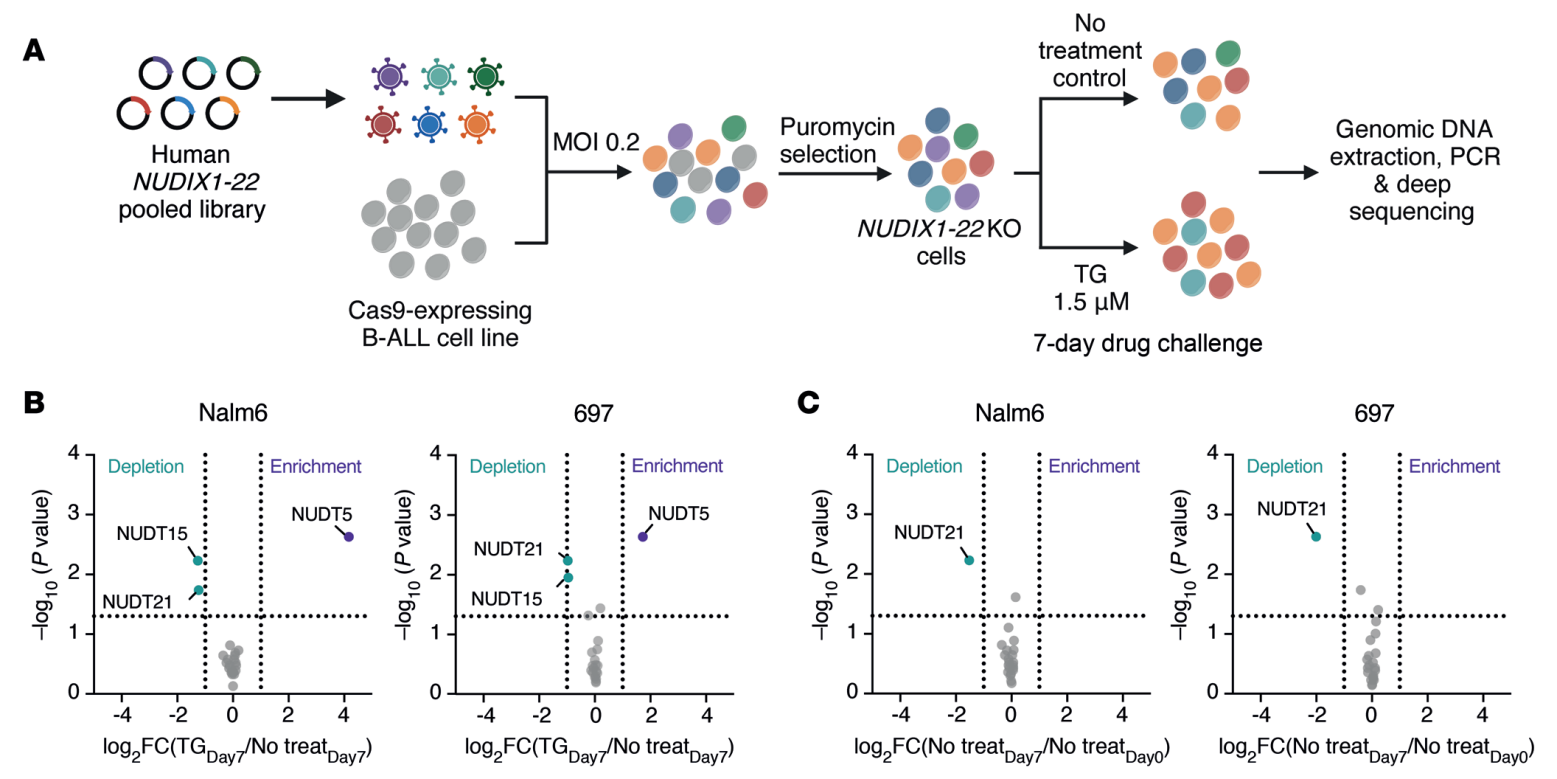
*NUDIX*-targeted CRISPR/Cas9 screen for modulators of thiopurine cytotoxicity. (**A**) Workflow for *NUDIX*-targeted CRISPR/Cas9 screen in B-ALL cell lines. Illustration was created with BioRender.com. (**B**) Volcano plot showing the enrichment or depletion of the sgRNAs targeting the respective *NUDIX* genes in CRISPR/Cas9-transduced cells after 7 days of TG (TG_Day7_) compared with control (No treat_Day7_). (**C**) Volcano plot showing enrichment or depletion of the *NUDIX* genes in CRISPR/Cas9-transduced cells after 7 days of culturing (No treat_Day7_) compared with day 0 of culturing (i.e., immediately after lentiviral transduction [No treat_Day0_]), without drug exposure. The *x* and *y* axes represent the FC (in logarithmic scale) and the nominal *P* value of the enrichment or depletion, respectively. The vertical dotted line indicates a log_2_(FC) of –1 or 1, and the horizontal dotted line represents a *P* value of 0.05.

To identify which of the 22 NUDIX hydrolases regulate the cytotoxic effects of thiopurines, we performed a *NUDIX*-targeted CRISPR/Cas9 screen in 2 human B-ALL cell lines, Nalm6 (*DUX4-IGH* rearranged B-ALL) and 697 (*TCF3-PBX1* fusion B-ALL) (Figure 1A). A library of 46 sgRNAs was assembled with 2 sgRNAs per gene, except *NUDT4*, for which 2 pairs of flanking sgRNAs were designed because of the small exon size. Following a 7-day exposure to TG, sgRNAs targeting *NUDT15* were significantly depleted, confirming the loss of this gene linked to thiopurine sensitivity (fold-change [FC] vs. no treatment control: 0.41 and 0.52, *P* = 0.006 and 0.011, in Nalm6 and 697 cells, respectively) (Figure 1B). Conversely, *NUDT5* sgRNAs were significantly enriched in B-ALL cells that survived thiopurine treatment (FC = 18.4 and 3.2, respectively, *P* = 0.002), indicating that NUDT5 deficiency drives drug resistance. The depletion of *NUDT21* in the control condition (without thiopurine) suggested it is essential for ALL survival (FC = 0.35 and 0.25, *P* = 0.006 and 0.002, respectively) (22) (Figure 1C). Using a FC of 2 or less or greater than 2 and a *P* value of less than 0.05 as the threshold of statistical significance, we identified no other *NUDIX* genes with significant effects on the thiopurine response.

### NUDT5 deletion results in marked thiopurine drug resistance.

**Figure 2:**
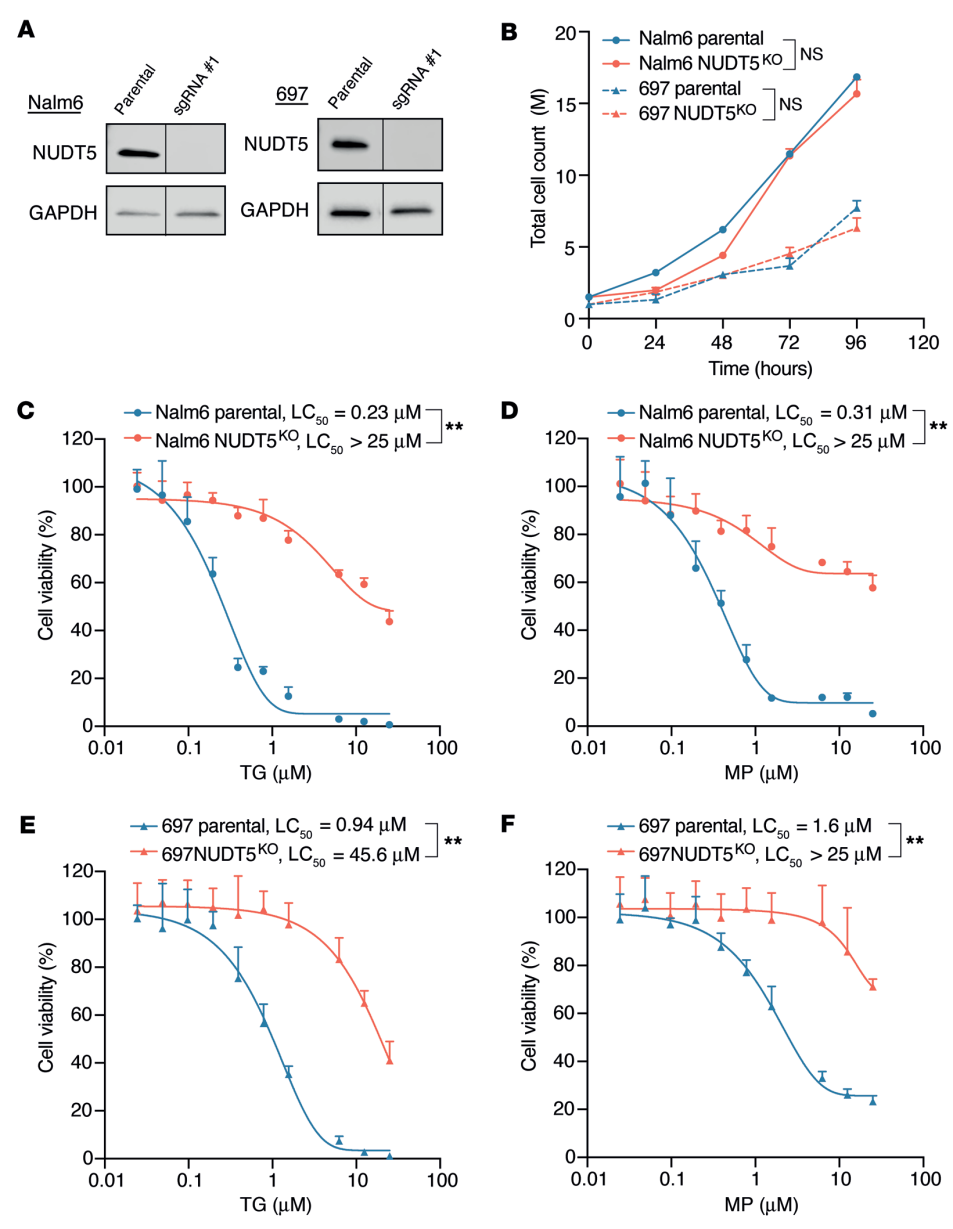
NUDT5 is required for thiopurine-induced apoptosis. (**A**) Western blots confirming *NUDT5* KO in the Nalm6 and 697 B-ALL cell lines. (**B**) Proliferation assay showing no difference in cell growth between *NUDT5*^KO^ and parental cell lines. (**C** and **D**) *NUDT5*^KO^ Nalm6 cell sensitivity to TG and MP, respectively, after 72 hours of treatment. (**E** and **F**) *NUDT5*^KO^ 697 cell sensitivity to TG and MP, respectively, after 72 hours of treatment. Data are presented as the mean ± SD. *n* = 3 replicates. **P < 0.01, by 2-tailed, unpaired *t* test for comparisons between groups. LC_50_, lethal concentration 50.

To characterize the molecular mechanism by which NUDT5 modulates the thiopurine response, we first generated *NUDT5*^KO^ B-ALL cell lines using one of the sgRNAs from the original CRISPR library and confirmed protein depletion by Western blotting (Figure 2A). In the absence of thiopurine drugs, *NUDT5* deletion did not alter ALL cell proliferation (Figure 2B). After exposing the cells to increasing concentrations of TG or MP, *NUDT5*-KO (*NUDT5*^KO^) Nalm6 cells showed minimal cell death, whereas both drugs were highly cytotoxic in parental cells in a dose-dependent manner (Figure 2, C and D). These results were replicated in the 697 cell line (Figure 2, E and F). Reexpression of *NUDT5* in the *NUDT5*^KO^ cells largely restored drug sensitivity (Supplemental Figure 1; supplemental material available online with this article; https://doi.org/10.1172/JCI190443DS1). As expected, the MMR-dependent DNA damage signaling pathway was activated in parental cells exposed to MP, e.g., histone H2AX phosphorylation and checkpoint kinase Chk1/Chk2 activation (23). However, no change was observed when *NUDT5* was knocked out, similar to the parental cells without MP treatment (Supplemental Figure 2).

### KO of NUDT5 compromises thiopurine biotransformation.

**Figure 3:**
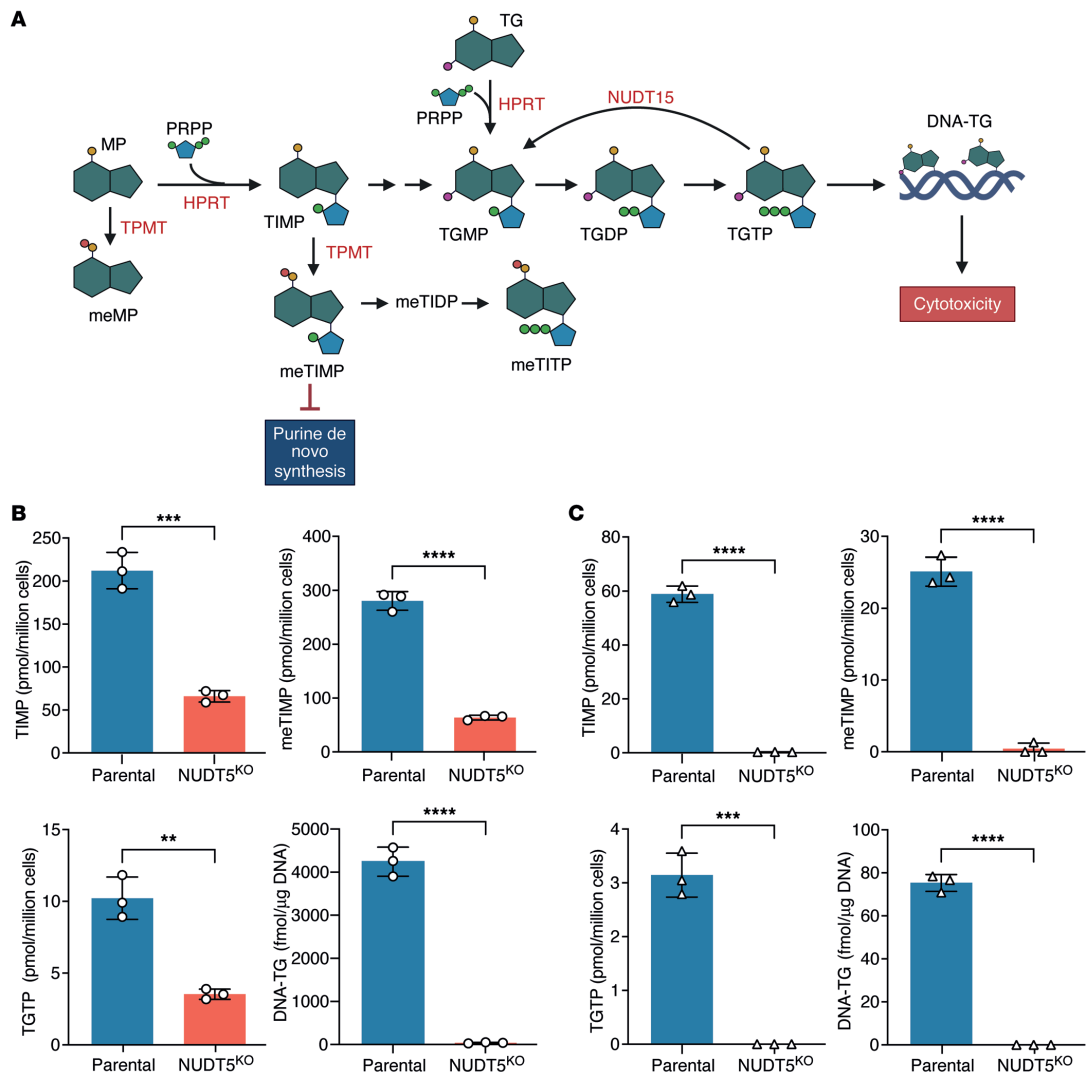
*NUDT5* deletion impairs intracellular metabolism of thiopurines. (**A**) Thiopurine metabolism pathway. Illustration was created with BioRender.com. (**B** and **C**) Cytosolic metabolites were measured in parental and *NUDT5*^KO^ Nalm6 (**B**) and 697 (**C**) cell lines after treatment with 10 μM MP for 24 hours. Nuclear DNA-TG was measured after treatment with 5 μM MP for 24 hours. Data are presented as the mean ± SD. *n* = 3 replicates. **P < 0.01. ***P < 0.001, and ****P < 0.0001, by 2-tailed, unpaired *t* test.

To determine whether thiopurine metabolism was altered in *NUDT5*^KO^ cells, we measured cytosolic concentrations of 12 thiopurine metabolites (namely, thioguanine mono-, di-, and triphosphate [TGMP, TGDP, and TGTP, respectively], thioinosine mono-, di-, and triphosphate [TIMP, TIDP, and TITP, respectively], and their respective methylated forms [meTGMP, meTGDP, meTGTP, meTIMP, meTIDP, and meTITP]), plus the degree of thioguanine incorporation into DNA (DNA-TG) (Figure 3A). The first product of MP biotransformation, TIMP, was significantly decreased in both *NUDT5*^KO^ cell lines (Nalm6: FC = 0.31, *P* < 0.001, 697: FC = 0.005, *P* < 0.001), as was its methylated species, meTIMP (FC = 0.23 and 0.017, *P* < 0.0001) (Figure 3, B and C). Similarly, the final product of thiopurine activation, TGTP, was significantly reduced in both *NUDT5*^KO^ cell lines (Nalm6: FC = 0.35, *P* = 0.0016, 697: not detected in *NUDT5*^KO^, *P* = 0.0002). Last, DNA-TG was almost completely absent in cells without NUDT5 (Nalm6: FC = 0.009, *P* < 0.001, 697: not detected in *NUDT5*^KO^, *P* < 0.0001). This depletion in DNA-TG was also observed when these cells were treated with TG, which utilizes a similar but not identical biotransformation pathway (Supplemental Figure 3). Together, these data indicate that NUDT5 is essential for the activation of thiopurines to exert their cytotoxicity.

### Depletion of NUDT5 inhibits the purine salvage pathway.

**Figure 4:**
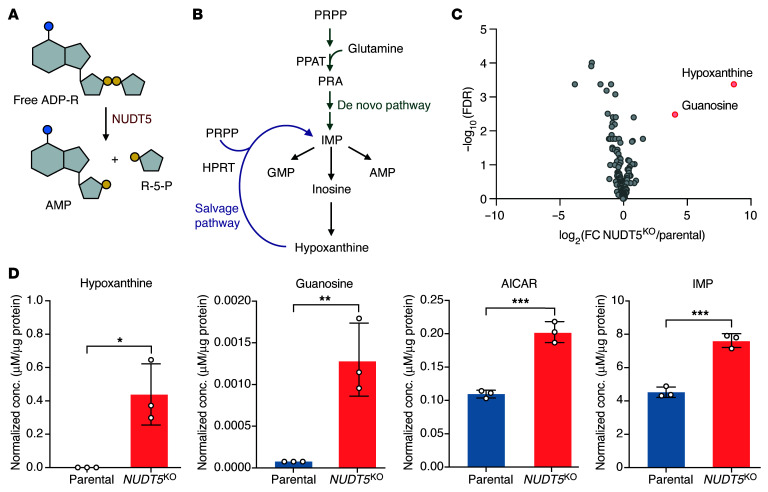
Targeted metabolomics profiling identifies the effects of NUDT5 on purine nucleotide homeostasis. (**A**) NUDT5 hydrolyzes ADP-R into AMP and R-5-P. (**B**) Illustration of de novo and salvage pathways for purine synthesis. (**C**) Volcano plot generated after data transformation and normalization of the concentrations of metabolites measured in Nalm6 parental and *NUDT5*^KO^ cells. (**D**) Levels of the purine intermediates hypoxanthine, guanosine, AICAR, and IMP in Nalm6 parental and *NUDT5*^KO^ cells. The concentrations (conc.) of the metabolites were normalized by the protein concentration in the sample. Data are represented as the mean ± SD. *n* = 3 replicates. *P < 0.05, **P < 0.01, and ***P < 0.001, by 2-tailed, unpaired *t* test. Illustrations in **A** and **B** were created with BioRender.com.

As a phosphatase, NUDT5 has been linked to the hydrolysis of adenosine diphosphate ribose (ADP-R) to generate ribose-5-phosphate (R-5-P) (Figure 4A), the precursor of phosphoribosyl pyrophosphate (PRPP) that is used for nucleotide biosynthesis, including both de novo and salvage purine synthesis (Figure 4B) (24). Building upon this, we first subjected both parental and *NUDT5*^KO^ cell lines to a broad metabolomics analysis examining 166 cellular metabolites involved in nucleotide, amino acid, and carbohydrate biosynthesis and degradation (Supplemental Table 1). The most significant change was seen with hypoxanthine and guanosine, 400-fold and 16-fold higher in *NUDT5*^KO^ compared with parental cells, respectively (*P* = 0.0004 and 0.003, Figure 4, C and D). Of the 23 purine-related metabolites detected, we also observed a significant increase of 5-aminoimidazole-4-carboxamide ribonucleotide (AICAR) and inosine monophosphate (IMP) in *NUDT5*^KO^ cells (2-fold, *P* = 0.0007, and 1.6-fold, *P* = 0.02, respectively). These changes pointed to the profound effect of *NUDT5* expression on purine metabolism.

**Figure 5:**
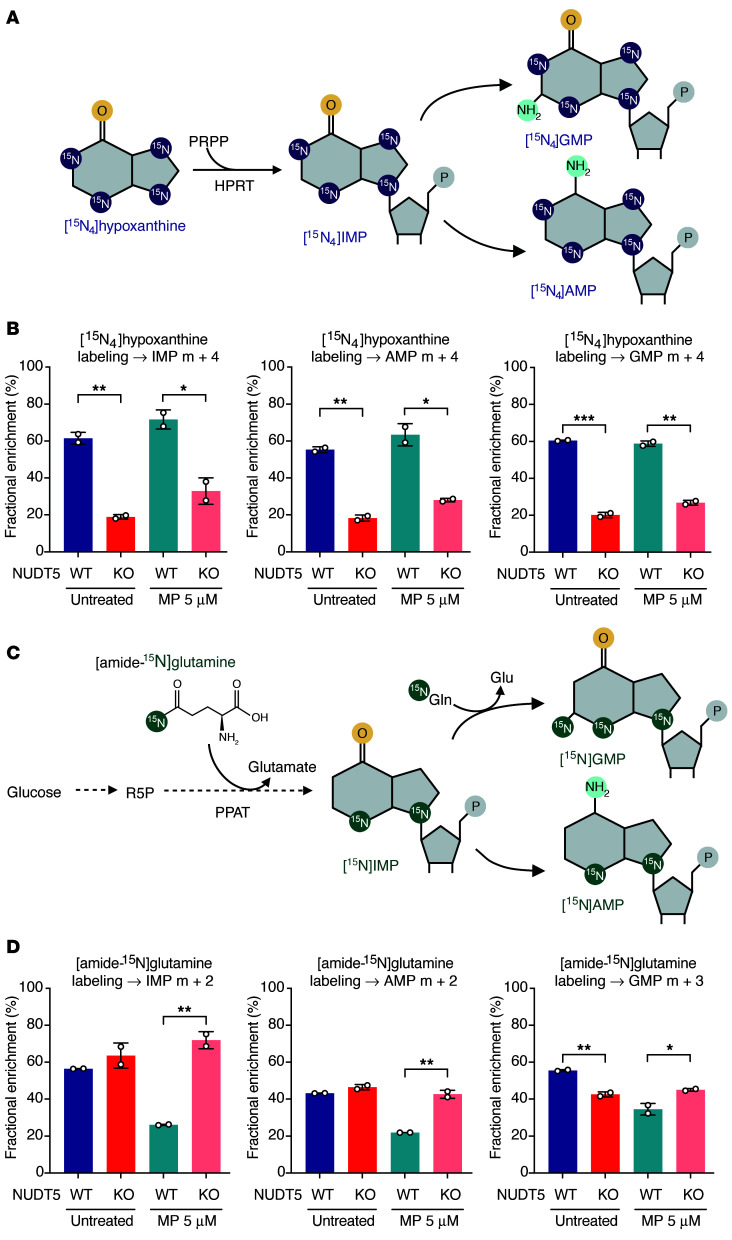
Stable isotope tracing of purine synthesis pathways highlights the contribution of NUDT5 to purine metabolism. (**A**) Schematic illustrating ^15^N labeling of purine nucleotides from [^15^N_4_]hypoxanthine during the purine salvage pathway. (**B**) Fractional enrichment of m+4 IMP, AMP, and GMP from [^15^N_4_]hypoxanthine in parental and *NUDT5*^KO^ Nalm6 cells during the purine salvage pathway, with and without MP. (**C**) Schematic illustrating ^15^N labeling of purine nucleotides from [amide-^15^N]glutamine during the de novo purine synthesis pathway. (**D**) Fractional enrichment of m+2 IMP and AMP, and m+3 GMP from [amide-^15^N]glutamine in parental and *NUDT5*^KO^ Nalm6 cells during the purine de novo synthesis, with and without MP. Data are presented as the mean ± SD. *n* = 2 replicates. *P < 0.05, **P < 0.01, and ***P < 0.001, by 2-tailed, unpaired *t* test. Panels **A** and **C** were created with BioRender.com.

To more precisely determine the role of NUDT5 in purine metabolism, we performed a series of isotope-tracing experiments focusing on metabolites related to either de novo purine synthesis (DNPS) or purine salvage pathways. Using nitrogen-labeled hypoxanthine ([^15^N_4_]hypoxanthine), we sought to track the ^15^N-labeled purine ring into IMP, guanosine monophosphate (GMP), and adenosine monophosphate (AMP) (m+4 labeling) through the salvage pathway (Figure 5A). As shown in Figure 5B, the loss of NUDT5 markedly reduced m+4 labeling of all purine nucleotides (all *P* < 0.05), consistent with downregulation of the salvage pathway. Exposure to MP had a minimal effect on these hypoxanthine-derived purine nucleotides in either WT (*P* = 0.14, 0.26, and 0.20, for IMP, GMP, and AMP, respectively) or *NUDT5*^KO^ (*P* = 0.11, 0.02, and 0.04, for IMP, GMP and AMP, respectively) cells. These results suggested that the first step of purine salvage, i.e., HPRT-catalyzed conversion of hypoxanthine to IMP, was impaired in *NUDT5*^KO^ cells. This was likely due to the loss of HPRT catalytic activity (or enzymatic inhibition), whereas *HPRT1* expression was unaffected in the absence of NUDT5 (Supplemental Figure 4).

In parallel, we examined DNPS using [amide-^15^N]glutamine, which is used by phosphoribosyl pyrophosphate amidotransferase (PPAT) to convert PRPP to phosphoribosylamine (PRA), the first precursor of purine nucleotides during DNPS (Figure 5C). Labeled glutamine provides two ^15^N atoms for the de novo synthesis of IMP, which is further converted to AMP (m+2 labeling) or to GMP using a second molecule of glutamine (m+3 labeling). As shown in Figure 5D, there was no change in m+2 labeling of IMP or AMP in *NUDT5*^KO^ cells relative to parental control cells, although we observed a modest decrease in m+3 labeling of GMP (*P* = 0.006), suggesting a minimal effect of NUDT5 on DNPS at baseline. In the presence of MP, we found that DNPS was greatly inhibited in parental cells, as reflected by the reduction of IMP, AMP, and GMP, whereas *NUDT5*^KO^ cells showed no MP-induced changes in DNPS.

Therefore, we reason that NUDT5 deficiency blunted HPRT activity, downregulating purine salvage and hampering the conversion of thiopurine to cytotoxic metabolites. By contrast, NUDT5 may have a minimal role in DNPS under physiological conditions but is required for DNPS inhibition by thiopurines.

### Association of NUDT5 germline variants with hematopoietic toxicity in patients receiving thiopurine therapy.

**Figure 6:**
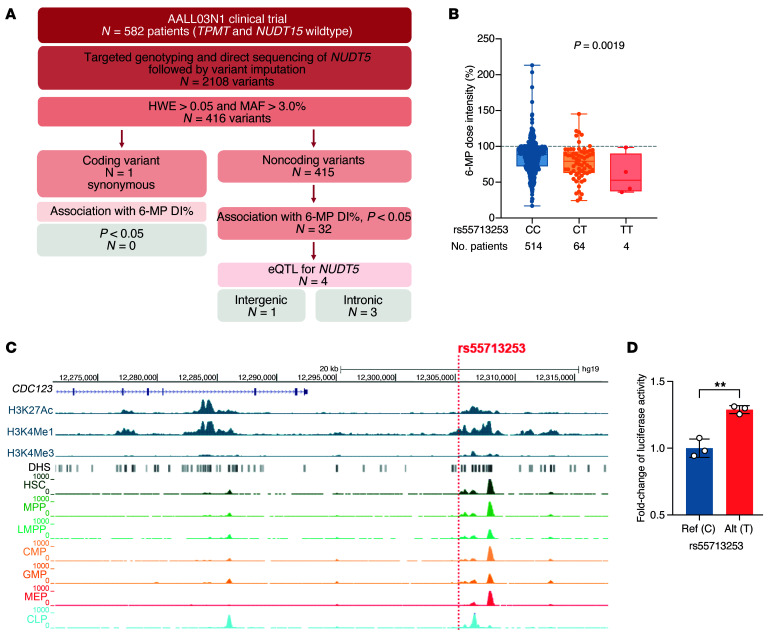
Characterization of *NUDT5* germline variants associated with thiopurine-induced myelosuppression in children with ALL. (**A**) *NUDT5* sequencing and genotyping data were retrieved for 582 children with ALL, who were enrolled in the Children’s Oncology Group AALL03N1 clinical trial. All patients were *TPMT* and *NUDT15* WT. Common variants (MAF >0.03) were considered for a multiple regression association analysis with MP sensitivity. HWE, Hardy-Weinberg Equilibrium. (**B**) The intergenic variant rs55713253 C>T on chromosome 10 was associated with a significant decrease in MP DI during maintenance therapy. Shown is the nominal *P* value of the multiple regression model, assuming an additive genetic model. (**C**) View of the genomic environment of rs55713253 C>T from the UCSC Genome Browser (https://genome.ucsc.edu/). CLP, common lymphoid progenitor; CMP, common myeloid progenitor; DHS, DNase hypersensitivity site; GMP, granulocyte-monocyte progenitor; HSC, hematopoietic stem cell; LMPP, lymphoid-primed multipotent progenitor cell; MEP, megakaryocyte-erythrocyte progenitor; MMP, multipotent progenitor. (**D**) Luciferase reporter gene assay in Nalm6 cells expressing the *NUDT5* rs55713253-mutant T allele showed increased expression of the reporter gene. Ref, reference allele; Alt, alternative allele. *n* = 3 replicates. Data are presented as the mean ± SD. **P < 0.01, by 2-tailed, unpaired *t* test.

To evaluate the clinical relevance of the NUDT5*-*mediated thiopurine response, we comprehensively characterized genetic variations of *NUDT5* in 582 children with ALL enrolled on the Children’s Oncology Group AALL03N1 trial, for whom systematic data on MP-related myelosuppression were collected (13). Patients with known pharmacogenetic variants in *TPMT* and *NUDT15* were excluded to avoid confounding effects. A total of 2,108 variants were identified within *NUDT5* exons and 100 kb upstream of the gene (Figure 6A). Among 416 common variants (minor allele frequency [MAF] >0.03), the only coding variant in *NUDT5* was a synonymous variant, rs6686 (c.609A>G, p.A203A), in exon 10, which was not associated with MP dose intensity (DI) (nominal *P* = 0.472).

Because *NUDT5* expression was linked to the thiopurine response, we hypothesized that *cis*-regulatory variants at this locus (i.e., expression quantitative trait loci, [eQTL], of *NUDT5*) are associated with MP dose intensity (DI, in %), a proxy marker for drug-induced myelosuppression. Among the 415 common noncoding variants, 32 were associated with MP DI during the maintenance therapy according to the multivariable regression model adjusting for genetic ancestry (*P* < 0.05), and 4 of them were related to *NUDT5* transcription in blood cells according to the public eQTLGen gene expression database (*P* < 0.0001) (Supplemental Table 2).

Only the intergenic variant rs55713253 (C>T, Chr10:12,305,358, MAF = 0.06 in the cohort) remained significant in a permutation-based analysis to account for multiple testing (nominal *P* = 0.0019 and adjusted *P* = 0.036). Patients with the WT genotype (CC, *n* = 514) tolerated a median DI of 89% (IQR, 72%–97.2%), which was higher than for patients heterozygous for rs55713253 (CT, *n* = 64, 79.2%, [63.1–92.7]), and the DI was the lowest in homozygous patients (TT, *n* = 4, DI = 52.7%, [37.4–90.0], Figure 6B). Consistent with our observation that NUDT5 potentiated thiopurine cytotoxicity, the T allele at this locus was also associated with elevated *NUDT5* expression in blood (*P* = 5.0 × 10^–19^, *z* score = 9.0, per eQTLGen) (25). We queried the RegulomeDB public database and found that rs55713253 was likely to affect *NUDT5* transcription in hematopoietic cells (probability score of 0.97 based on chromatin accessibility and transcription factor binding data); it occurs 67 kb upstream of the *NUDT5* transcription start site, in a region enriched in histone 3 methylation marks (H3K4Me1 and H3K4Me3) (Figure 6C). This variant overlaps with an YY1 ChIP-Seq peak in GM12878 lymphoblastoid cells according to the Encyclopedia of DNA Elements (ENCODE) database. We experimentally tested the effect of rs55713253 on enhancer activity with a reporter gene assay and found that the T allele significantly increased luciferase expression, confirming its potential to influence *NUDT5* expression (FC = 1.29, *P* = 0.01, Figure 6D).

## Discussion

In this study, we comprehensively explored the role of NUDIX hydrolases on thiopurine metabolism, efficacy, and toxicity. Our *NUDIX*-targeted CRISPR/Cas9 screen confirmed NUDT15 as a negative regulator of cytotoxicity of this class of drugs, but, surprisingly, we discovered that NUDT5 was required for thiopurine-induced cell death. In fact, several prior studies have reported similar findings, so much so that NUDT5 was the top thiopurine resistance gene in a genome-wide fashion (26), although the underlying biology is not understood. Physiologically, NUDT5 plays a critical role in metabolic homeostasis by hydrolyzing cytosolic ADP-R into AMP and R-5-P (24, 27–29). Because R-5-P is the precursor of PRPP, which is required for the conversion of MP to TIMP or TG to TGMP (24, 28), we initially hypothesized that NUDT5 deletion causes the loss of PRPP and thus impairs thiopurine biotransformation (Figure 4A). A few lines of evidence from us and others seem to refute this hypothesis: (a) leukemia cells generally favor the pentose phosphate pathway to produce R-5-P from glucose (30), and NUDT5-mediated hydrolysis of ADP-R is probably a minor source for this metabolite; (b) NUDT5 deletion specifically resulted in drastic reduction of purine salvage without affecting DNPS (Figure 5), even though both pathways depend on PRPP. Instead, our data suggest that the loss of NUDT5 impaired HPRT function (directly or indirectly), which limited the cell’s ability to convert thiopurine prodrug into active metabolites (TIMP, TGTP, and DNA-TG) and, in turn, reduced drug cytotoxicity. However, the exact molecular process by which NUDT5 modulates HPRT activity and purine salvage in this context remains unclear.

It should not go unnoticed that NUDT5 deletion also led to nearly complete depletion of meTIMP, the thiopurine metabolite responsible for DNPS inhibition (31, 32). These data raise the question of whether thiopurines exert their cytotoxic effects by being incorporated into nucleic acids and causing DNA mismatch (historically considered the major toxic pathway), or by inhibiting DNPS, previously deemed to be a minor pathway (33). The fact that *NUDT5* KO also led to resistance to TG (which does not affect DNPS) points to DNA damage as the main cause of thiopurine-induced cell death in our model systems. Nonetheless, NUDT5 clearly contributed to thiopurine activation, regardless of the exact pathways leading to cytotoxicity. Thiopurine cytotoxicity and the rate of DNPS both vary substantially by ALL subtype (34, 35), but this is not likely to be related to *NUDT5* because its expression is relatively consistent across ALL subtypes (St. Jude Cloud, ALL expression dataset of 925 cases: https://pecan.stjude.cloud/expression/gene-expression), However, we cannot completely rule out the subtype-dependent effects of NUDT5 on thiopurine metabolism.

Our comprehensive analysis of *NUDT5* genetics in children with ALL suggests that increased expression of *NUDT5* may be linked to reduced tolerance to thiopurines. In this context, higher enzymatic activity would be associated with increased thiopurine activation, leading to elevated TGNs and a higher risk of hematopoietic toxicity. For example, 3 of the 4 patients homozygous at rs55713253 (TT genotype) required a greater than 50% dose reduction, with a median DI lower than that in patients with the CT genotype, although this difference did not reach statistical significance plausibly because of our limited sample size. Therefore, we reason that *NUDT5* variants may have a sizable effect on thiopurine tolerance but that the effects seem more variable than what we have seen with *NUDT15* and *TPMT* variants. Assuming an additive allelic effect, *TPMT* variants (rs1142345, rs1800462, rs1800460) were estimated to explain 4.9% of the interpatient variability in DI in our study cohort, whereas *NUDT15* rs116855232 would explain 6.1% of it. By comparison, the *NUDT5* variant (rs55713253) explains an additional 1.8% variability in this phenotype. Larger cohorts will be needed in the future to define the precise clinical relevance of the *NUDT5* genotype in thiopurine dosing. Finally, our findings also suggest that somatic loss-of-function mutations in *NUDT5* could lead to thiopurine resistance, potentially reducing the efficacy of ALL therapy. However, when we queried whole-genome or whole-exome sequencing data on 2,754 ALL cases, we found no *NUDT5* mutations in leukemia genomes (36).

In conclusion, we identified NUDT5 as a key modulator of thiopurine metabolism and thus of efficacy and/or toxicity, and our mechanistic studies provided insights into NUDT5-mediated regulation of purine homeostasis. Our findings indicate that the efficacy of thiopurines may be compromised in patients with *NUDT5* deficiency, whereas *NUDT5* activation can result in excessive toxicity, pointing to the importance of *NUDT5* in thiopurine pharmacogenomics.

## Methods

### Sex as a biological variable.

Our study examined male and female patients. Details about the cohort have been previously published (37).

### NUDIX-targeted CRISPR/Cas9-KO screen.

A library of 46 sgRNAs targeting 22 *NUDIX* genes was designed and purchased from MilliporeSigma, with each gene covered by 2 guides, except for *NUDT4*, for which 2 pairs of flanking sgRNAs were designed due to the small exon size (Supplemental Table 3). The Cas9-expressing human B-ALL cell lines Nalm6 and 697 were used for lentiviral transduction. Cells were cultured in RPMI-1640 (Gibco, Thermo Fisher Scientific, 11875093) containing 10% FBS (Cytiva, SH30071.03) at 37°C with 5% CO_2_. Transduced cells with sgRNAs were selected with puromycin and challenged with 1.5 μM TG for 7 days. The sgRNA sequences were recovered by genomic PCR and detected by deep sequencing performed on a MiSeq system (Illumina) (38). Sequencing data were analyzed with MAGeCK, version 0.5.9.5 (39). Details on the CRISPR/Cas9 screen can be found in the Supplemental Methods.

### CRISPR/Cas9 genome editing of NUDT5.

One of the sgRNAs of the pair targeting *NUDT5* in the CRISPR/Cas9-KO library (sgRNA 1, 5′-CACCGGAAGTGTTCTCTGCAGCACG-3′) was subcloned into the pSpCas9(BB)-2A-GFP (px458) vector (Addgene, 48138). The B-ALL cell lines Nalm6 (American Type Culture Collection [ATCC], CRL-3273) and 697 (DSMZ, ACC42) were a gift from William E. Evans (St. Jude Children’s Research Hospital, Memphis, Tennessee, USA). The cell lines were transfected by electroporation using the Cell Line Nucleofector Kit R (Lonza, VCA-1001) and the Nucleofector II device (Lonza). Single cells were sorted by flow cytometry 48 hours after transfection on the basis of size, viability, and GFP^+^ expression using a Bigfoot Spectral Cell Sorter (Invitrogen, Thermo Fisher Scientific) and the LSRFortessa Cytometer (BD Biosciences). Flow Jo software, version 10.8.1, was used for data analysis. Single clones were selected and cultured using semi-solid ClonaCell-TCS Medium (STEMCELL Technologies, 03814). KO of *NUDT5* was verified by Western blotting (see corresponding section for details).

### NUDT5 overexpression in KO cells.

*NUDT5* cDNA was amplified by PCR and subcloned into a cl20c-IRES-mCherry lentiviral plasmid (40) using the NEBuilder HiFi DNA Assembly Cloning kit (New England Biolabs [NEB], E5520) (Supplemental Table 4). Purified empty vector and cl20c-NUDT5-IRES-mCherry plasmids were respectively transfected together with packaging vectors into Lenti-X 293T cells (Takara, 632180). After 48 hours of transfection, the culture media containing lentivirus particles were collected to infect the Nalm6 *NUDT5*^KO^ cell line. Transduction efficiency was determined after 48 hours using flow cytometry, and mCherry^+^ cells were sorted, as described above.

### In vitro thiopurine sensitivity assessment.

Sensitivity to thiopurine was tested in *NUDT5*^KO^ cell lines and *NUDT5*-overexpressing Nalm6 cells. Briefly, cells were seeded into a 384-well microplate with 1,250 cells per well, and gradient-diluted MP or TG was added to the microwells. After 72 hours of incubation, cell viability was measured by the CellTiter-Glo assay (Promega, G8461), and luminescence was detected with the Synergy H4 hybrid reader (Agilent BioTek).

### Western blotting.

A total of 1 million cells were harvested and washed once with cold PBS (Gibco, Thermo Fisher Scientific, 10010023). Cell pellets were lysed with 100 μL RIPA lysis buffer (Thermo Fisher Scientific, J63306.AP). The protein concentration in the lysates was measured using the BCA assay kit (Thermo Fisher Scientific, 23227). Protein (30 μg) for each sample was subjected to SDS-PAGE, followed by membrane transfer and incubation with antibodies. The following monoclonal antibodies for the detection of proteins of interest and control proteins were purchased from Abcam or Cell Signaling Technology (CST): β-actin (CST, 4967), GAPDH (CST, 14C10), HPRT (Abcam, EPR5299), NUDT5 (Abcam, EPR7734), pATM (CST, D6H9), pATR (CST, D5K8W), pChk1 (CST, 133D3), pChk2 (CST, E8Q1A), and γH2AX (CST, 2577). The detection was revealed using a secondary antibody conjugated to horseradish peroxidase (HRP) (Anti-rabbit IgG, Amersham ECL HRP-linked Antibody NA9340, Cytiva).

### Measurement of thiopurine cytosolic and nucleic metabolites.

Twelve thiopurine cytosolic metabolites (thioguanine nucleotides: TGMP, TGDP, TGTP; methyl thioguanine nucleotides: meTGMP, meTGDP, meTGTP; thioinosine nucleotides: TIMP, TIDP, TITP; methylthioinosine nucleotides: meTIMP, meTIDP, meTITP) were extracted from Nalm6 and 697 *NUDT5*^KO^ cells after 24 hours of MP (10 μM) and analyzed by liquid chromatography coupled to tandem mass spectrometry (LC-MS/MS) according to a previously published method (41). For the quantification of nuclear DNA-TG, genomic DNA was extracted from cells similarly exposed to thiopurines using the DNeasy Blood and Tissue kit (Qiagen, 69506). Eluted DNA was digested for 24 hours at 37°C with a cocktail of enzymes (all purchased from MilliporeSigma) containing benzonase (E1014), phosphodiesterase I (P3243), and alkaline phosphatase (P7923) mixed in a solution of Tris-HCl (20 mM, pH 7.9) with 100 mM NaCl and 20 mM MgCl_2_ (Invitrogen). DNA-TG was detected and quantified by LC-MS/MS following a procedure adapted from previously published methods (42, 43).

### Metabolomics analysis.

To evaluate the effect of NUDT5 deletion on endogenous metabolite homeostasis, we performed steady-state metabolomics profiling with Nalm6 parental and *NUDT5*^KO^ cell lines. Briefly, 5 million cells were harvested by centrifugation and washed twice with ice-cold PBS, and the pellets were snap-frozen in liquid nitrogen to quench intracellular metabolism. Endogenous metabolites were extracted according to a previously described method (44). Cell extracts were subjected to LC/MS-MS–based targeted metabolomics analyses. Metabolite concentrations were normalized to the protein content of each sample. The metabolomics data were analyzed with Metaboanalyst, version 6.0 (45). For the univariate analysis, metabolite concentrations were normalized to the median of replicates and were log transformed. For the enrichment analysis, metabolite abundance was compared with the Kyoto Encyclopedia of Genes and Genomes (KEGG) human metabolic pathways.

### Stable isotope tracing in B-ALL cells.

Stable isotope tracing assays were performed in Nalm6 parental and Nalm6 *NUDT5*^KO^ cells. All stable isotopes were purchased from Cambridge Isotope Laboratories. For the hypoxanthine tracing assay, 10 million cells were cultured in base RPMI (MilliporeSigma, 11875-093) with 10% of dialyzed FBS (GeminiBio, 100-108) and supplemented with 10 μM [^15^N_4_]hypoxanthine (NLM-8500-0.1, Cambridge Isotope Laboratories). For the glutamine tracing experiment, 10 million cells were incubated in glutamine-free RPMI (MilliporeSigma, R0883) with 10% dialyzed FBS and supplemented with 2 mM [amide-^15^N]glutamine (NLM-557-1, Cambridge Isotope Laboratories). Metabolite extraction was performed after 4 hours of incubation in the respective labeled medium, according to a previously published method (46). Briefly, cell pellets were rinsed with ice-cold saline twice and quenched by 80% cold methanol. Samples were subjected to three freeze-thaw cycles using liquid nitrogen and a 37°C water bath and then homogenized using a vortex mixer. Samples were then centrifuged at 20,000*g* for 15 minutes at 4°C, and the supernatants were collected into new tubes and dried overnight in a SpeedVac concentrator. Dried metabolites were reconstituted in analytical-grade water containing 0.1% formic acid. Samples were analyzed using reverse-phase liquid chromatography coupled with a high-resolution MS Q-TOF mass spectrometer (46).

### Patients and thiopurine therapy.

Clinical data and biological specimens were collected for 582 patients with ALL enrolled in the Children’s Oncology Group’s AALL03N1 clinical trial (37). All patients received daily thiopurine for the maintenance phase of their ALL. Dose adjustments were based on the monitoring of hematopoietic function and the occurrence of severe infections. MP DI (in percentages) was used as a surrogate marker of thiopurine-related toxicity, as previously described (13).

### NUDT5 sequencing and genotyping in patients.

Germline DNA was extracted from peripheral blood or bone marrow obtained during clinical remission, and samples were submitted for targeted sequencing of exonic regions of *TPMT*, *NUDT15*, and *NUDT5*, in addition to genotyping by genome-wide mapping array (Affymetrix SNP6 Mapping array), as previously described (13, 47, 48). Additional genotypes were imputed using the TOPMed imputation server. Only patients with *TPMT* and *NUDT15* WT genotypes were included in this study.

### Association of NUDT5 variants with thiopurine toxicity.

A univariate linear regression model was used to describe the association between patients’ demographics and MP DI. A multiple regression model was applied to evaluate the association between *NUDT5* genetic variants and MP DI, with population structure included as a covariate (13). Age at diagnosis, WBC count, and sex were not statistically significantly associated with MP DI (*P* > 0.05) and thus not adjusted for in the regression model. We assumed an additive genetic model, in which genotype was treated as an ordinal variable on the basis of the number of copies of the alternative allele. Associations with a nominal *P* value of less than 0.05 were considered statistically significant, and permutation was used to adjust for multiple testing (Supplemental Methods) to account for linkage disequilibrium among SNPs. The eQTLGen public gene expression database was used to perform the *cis*-eQTL analysis (49). Only variants located at a putative enhancer region according to the HaploReg, version 4, database were included in the permutation-based analysis to account for multi-testing (50). In each permutation, DIs were randomly assigned to patients, and the association between the permuted DIs and genotypes was assessed using the same regression model for each candidate SNP. The smallest *P* value was recorded for each permutation. The adjusted *P* value was the number of permuted *P* values less than or equal to the observed *P* value. A total of 1,000 permutations were performed. The effect of the variants of interest on gene transcription was assessed using the RegulomeDB database, version 2.2 (51).

### Dual-luciferase reporter assay.

A 300 bp sequence centered on the reference or alternative allele of intergenic variant rs55713253 (chromosome 10:12305258, hg19; C>T) was subcloned upstream of the minimal promoter into the pGL4.23-basic vector using EcoRV restriction sites and validated by Sanger sequencing (see Supplemental Table 4 for primers). pGL4.23 luciferase plasmid DNA (60 μg) and a pRL-TK *Renilla* control vector (6 μg) were introduced into Nalm6 cells (10 million cells per replicate) using the Neon Transfection system (100 μL tip, Thermo Fisher Scientific, MPK5000). The transfection parameters were: 1,600 V, a width of 20 ms, and 1 pulse. The activity of firefly luciferase and *Renilla* was determined 48 hours after transfection using the Dual Luciferase Reporter Assay system (Promega, E1960) on a BioTek Cytation1 cell imaging multimode reader (Agilent Technologies). Luciferase activity was determined by normalizing Firefly luciferase signal to *Renilla* signal.

### Statistics.

All statistical tests were 2 sided and chosen as appropriate according to data distribution. A *P* value of less 0.05 was considered statistically significant. All *P* values are presented as nominal unless otherwise specified. MP DI was compared between *NUDT5* variant genotypes with the nonparametric Wilcoxon rank-sum test. The association study with *NUDT5* variants was performed with the rvtest package (52) for R software, version 4.1.3. Data were plotted with GraphPad Prism, version 10.0.0 (GraphPad Software). Data are presented as the mean ± SD.

### Study approval.

The human subject research was approved by the respective IRBs, those of St. Jude Children’s Research Hospital and the Children’s Oncology Group–affiliated institutions. Written informed consent (or assent when appropriate) were obtained from the parents, guardians, and/or patients. This study was approved by the IRB of St. Jude Children’s Research Hospital and was conducted in accordance with Declaration of Helsinki principles.

### Data availability.

Values for all data points in graphs are reported in the Supporting Data Values file. Genomics data and dose intensity data for the AALL03N1 cohort were deposited in the Database of Genotypes and Phenotypes (dbGaP) (study accession: phs002846.v1.p1).

## Author contributions

MM and RN are co–first authors, as they both contributed to the acquisition, analysis, and interpretation of data, with MM listed first since she drafted and revised the manuscript. JJY conceived and designed the project. MM, RN, HSV, KRB, JL, and UH performed the experiments. WY analyzed the genomics data. MM, RN, WY, DS, SB, MS, MN, and JJY interpreted the data. MM, WY, MN, and JJY contributed to the writing of the manuscript. All authors reviewed and agreed to the definitive version of the manuscript.

## Supplementary Material

Supplemental data

Unedited blot and gel images

Supporting data values

## Version 1 07/15/2025

Electronic publication
