# Supplementary material for: The NUDIX hydrolase NUDT5 regulates thiopurine metabolism and cytotoxicity
Source: J Clin Invest. 2025 Jul 15;135(14):e190443. doi: 10.1172/JCI190443 (PMC12259257; doi:10.1172/JCI190443)
Supplement: Supplemental data [file jci-135-190443-s072.pdf]

The NUDIX hydrolase NUDT5 regulates thiopurine metabolism and cytotoxicity

Supplemental Methods

Supplemental Table 1: Results of the metabolomics analysis in parental and *NUDT5*^{KO} cells.

Supplemental Table 2: Common non-coding variants associated with 6-mercaptopurine dose intensity in patients with acute lymphoblastic leukemia and eQTL for *NUDT5*.

Supplemental Table 3: Single guide RNA sequences designed for the *NUDIX*-targeted CRISPR/Cas9 screen.

Supplemental Table 4: List of primers.

Supplemental Figure 1: The overexpression of *NUDT5* in NUDT5 depleted B-acute lymphoblastic leukemia cells restores thiopurine sensitivity.

Supplemental Figure 2: NUDT5 depletion abolishes the activation of DNA damage response pathway after treatment with 6-mercaptopurine.

Supplemental Figure 3: NUDT5 disruption alters the incorporation of thiopurine active metabolite into DNA after treatment with 6-thioguanine.

Supplemental Figure 4: *HPRT1* expression in *NUDT5* knockout cells.

Supplemental Methods

CRISPR/Cas9 screen

A library of 46 sgRNAs targeting the twenty-two *NUDIX* genes was designed and purchased from Millipore Sigma (**Supplemental Table 3**). Two sgRNAs were selected per gene except for *NUDT4* for which two pairs of flanking sgRNAs were designed due to small exon size. Lentivirus was produced by transient transfection into Lenti-X HEK293T packaging cells (Takara, # 632180) with polyethyleneimine (Millipore Sigma). The Nalm6-Cas9 expressing cell line was transduced with low multiplicity-of-infection (MOI < 0.2) to achieve a representation of at least 500 cells per sgRNA. Cells were selected 24 hours after transduction by adding puromycin (5 µg/ml). After recovery, successfully transduced cells were treated for 7 days with 1.5 µM 6-thioguanine (TG). After treatment, genomic DNA was extracted using the Blood and Cell Culture Maxi kit (Qiagen, #13362) per manufacturer's protocol. The sgRNA region was amplified by PCR with dual indexed primers followed by sequencing via Illumina MiSeq for paired-end 100-bp read length. The sgRNA reads count and hits calling were analyzed by the Model-based Analysis of Genome-wide CRISPR-Cas9 Knockout (MAGeCK) algorithm tools available on the Galaxy server (version 0.5.9.5, https://usegalaxy.eu/) (1). Log2 fold changes (Log_2FC) and corresponding *P* values were used for the comparison between tested and control conditions.

Genomics analysis of *NUDT5* in patients with ALL

For targeted *NUDT5* sequencing, germline DNA was subjected to the generation of Illumina dual-indexed libraries, as previously described (2). All exons of *NUDT5* were captured by pooling the libraries in sets of 96 and were hybridized with customized Roche NimbleGene SeqCap EZ probes (Roche, Roche NimbleGen, Madison, Wisconsin, USA). Quantitative PCR was performed to define the appropriate capture product amplification to efficiently populate an Illumina HiSeq

2000 flow cell for paired end sequencing. Sequencing reads in FASTQ format were mapped and aligned by using the Burrows–Wheeler Aligner, and genetic variations were called by using GATK pipeline, as previously described (3). Only those with genotypes passing GATK quality control and exhibiting call rates greater than 95% were included in further association analyses. Across samples, a depth coverage of greater than 20x was achieved for more than 90% of the targeted regions. Additionally, samples were subjected to genotyping by using the Affymetrix Mapping SNP6 array. We further imputed patient genotypes using TopMed imputation server. Variants found within *NUDT5* or within 100K base pairs upstream of *NUDT5* transcription start site were tested for their association with thiopurine sensitivity.

Association of *NUDT5* genetic variants and MP sensitivity in patients with ALL

An additive multiple regression model was used to evaluate the association between *NUDT5* genetic variants and MP dose intensity (DI) during maintenance therapy. Each variant's genotype was considered as a continuous variable and coded as 0,1 or 2 based on the number of copies of the variant allele carried by the individual. Population structure was included as covariate. In each permutation step, we randomly shuffled the patients' phenotype, and the same multiple regression was performed for each variant. The adjusted *P* value is the number of times the observed *P* value is equal or less than those generated by permutation. A total of 1000 permutations were performed. A *cis*-eQTL analysis was conducted by retrieving variants from the public database eQTLGen (4) and variants with a *P* value < 0.0001 were considered eQTL for *NUDT5*. SNPs were only included in the permutation analysis if they were located at putative enhancer region according to HaploReg v.4 database (5).

Supplemental Table 1: Results of the metabolomics analysis in parental and *NUDT5*^{KO} cells.

Metabolite	log2(Fold-Change *NUDT5*^{KO}/parental cell line)	Adjusted P-value (False Discovery Rate)
5-Hydroxyindoleacetic acid	-2.4957	0.0000981
Linoleyl Carnitine	-2.5442	0.00012417
Hypoxanthine	8.6614	0.00042292
Myristoyl-L-carnitine	-3.8289	0.00042292
Palmitoyl-L carnitine	-1.8204	0.00042292
Beta-nicotinamide D-ribonucleotide	-0.97857	0.00042292
Uridine diphosphate glucose	-1.3422	0.0006499
Uracil	-0.64962	0.00083725
Uridine diphosphate	-1.2501	0.0031108
Guanosine	4.0345	0.003295
Uridine diphosphate-N-acetylglucosamine	-0.83362	0.0039466
Uridine	-0.61116	0.0039466
5-Aminoimidazole-4-carboxamide ribonucleotide (AICAR)	0.79477	0.0039832
Creatine	-0.8662	0.0058391
Cytidine	-0.6931	0.006482
Propionyl-L-carnitine	-0.93111	0.0075318
Cytidine diphosphate	-0.93718	0.013313
Acetyl-CoA	1.5198	0.017262
Nicotinic acid mononucleotide	-1.1472	0.017262
Uridine 5'-monophosphate	-1.0484	0.017262
Agmatine	-0.84985	0.017262
Hippurate	-0.64604	0.017262
Taurine	-0.36518	0.017262
AICA-riboside	0.71869	0.019853
Inosine monophosphate	0.66023	0.020872
Xanthylic acid	-0.25386	0.022378
Uridine triphosphate	-0.87821	0.031057
L-Dihydroorotic acid	-0.8865	0.034184
Cytidine monophosphate	-0.67835	0.035835
Cytosine	-0.47809	0.035835
Dihydroxyacetone phosphate	-0.54191	0.041532
Betaine	-0.40107	0.04819
3-Hydroxy-3-methylglutaryl-CoA	-0.30695	0.062979
D-Citramalic acid	-0.23836	0.067911
Adenosine triphosphate	0.35701	0.072896
Succinic acid	0.4489	0.076152
Biotin	-0.51421	0.079125
Pantothenic acid	-0.23824	0.086117
dCTP	0.86087	0.09537
Xanthosine	0.69081	0.09537
D-Ribose 5-phosphate	-1.055	0.10171
NADP	-0.68638	0.10171
2-ketoglutaric acid	-0.33939	0.10171
Choline	0.47506	0.1158
L-Homocysteine	-0.40179	0.11698

Supplemental Table 1: Results of the metabolomics analysis in parental and *NUDT5*^{KO} cells (continued).

Metabolite	log2(Fold-Change *NUDT5*^{KO}/parental cell line)	Adjusted P-value (False Discovery Rate)
Putrescine	0.40709	0.13071
N-Methyl-D-aspartic acid	0.31251	0.13649
Riboflavin	0.4068	0.14307
Gamma-Aminobutyric acid	-0.47989	0.1663
N-Acetyl-D-glucosamine	-0.4292	0.1663
L-Aspartic acid	0.41447	0.1736
Citrulline	-0.43929	0.1816
dCMP	-0.33636	0.1816
NAD	-0.31168	0.19759
Glutamine_2	0.53595	0.21126
Flavin Mononucleotide	-0.50941	0.21126
L-Pipecolic acid	0.4522	0.21145
S-Adenosylmethionine	-0.3948	0.21145
Dimethylglycine	0.27732	0.21145
N-Phenylacetylglycine	-0.23589	0.23307
Citicoline	0.27667	0.24617
Glucuronic acid	-0.42196	0.25424
L-Kynurenine	-0.22464	0.25424
Malonyl-L-carnitine	-1.1174	0.26268
Serine	-0.40307	0.26943
Thiamine	0.22707	0.26943
dAMP	-0.34371	0.27224
Cystathionine	0.28424	0.27737
N-Acetylputrescine	-0.26672	0.27737
Imidazole	0.97273	0.29403
N-Acetyl-glucosamine 1-phosphate	-0.6043	0.31154
Pyridoxal 5'-phosphate	-0.30945	0.31154
Adenine	-0.25316	0.31154
Lysine	-0.22388	0.31154
N-Acetylornithine	-0.20514	0.31154
Glutathione	-0.15201	0.31154
Asymmetric dimethylarginine	-0.19998	0.31554
Creatinine	-0.22531	0.32153
Sucrose	0.8356	0.34658
Deoxyuridine	0.54009	0.34658
dTMP	-0.30432	0.34658
S-Adenosylhomocysteine	-0.26558	0.34658
Arginine	-0.24671	0.34658
Gluconic acid	-0.22869	0.34658
Valeryl-L carnitine	-0.22352	0.34658
cis aconitic acid	-0.21621	0.34658
2-Ketohexanoic acid	-0.15001	0.34658
L-Glutamic acid	0.22749	0.34874
pyridoxine	-0.40653	0.36839

Supplemental Table 1: Results of the metabolomics analysis in parental and *NUDT5*^{KO} cells (continued).

Metabolite	log2(Fold-Change *NUDT5*^{KO}/parental cell line)	Adjusted P-value (False Discovery Rate)
N-Acetylglutamine	-0.21668	0.36839
Sorbitol	-0.18316	0.36839
GDP	-0.14027	0.36839
L-Acetylcarnitine	-0.13922	0.36839
Glucose 1-phosphate	-0.22407	0.37196
L-Proline	-0.18815	0.38482
Xanthine	-0.53849	0.38525
Phenyllactic acid	-0.098292	0.39858
Histidine	-0.086132	0.39858
dTTP	-0.26861	0.40406
N-Alpha-acetyllysine	-0.17414	0.42428
Carnitine	0.11528	0.44287
GMP	-0.17005	0.47261
Argininosuccinic acid	-0.22944	0.48377
dGDP	-0.18389	0.49451
N-Acetyl-L-alanine	0.19831	0.50196
Imidazoleacetic acid	-0.18827	0.50196
dGMP	0.14962	0.50196
Urea	-0.12853	0.50196
Adenosine	-0.21359	0.50989
L-Malic acid	0.07558	0.51502
dATP	0.25364	0.53785
Phosphorylcholine	0.19527	0.54477
L-Asparagine	-0.10347	0.54477
4-Hydroxyproline	-0.077474	0.58785
Kynurenic acid	-0.23075	0.60512
Deoxyribose 5-phosphate	0.10266	0.60512
Taurodeoxycholic acid	-0.5132	0.62435
D-Glyceraldehyde 3-phosphate	0.23021	0.65451
Spermidine	-0.22547	0.65451
Cyclic AMP	-0.20888	0.67232
Picolinic acid	-0.1569	0.67232
Leucine	0.13548	0.67232
L-Alanine	-0.074475	0.67232
CDP-ethanolamine	0.071345	0.67232
AMP	0.10197	0.67383
Nicotinamide	-0.14851	0.68004

Supplemental Table 1: Results of the metabolomics analysis in parental and *NUDT5*^{KO} cells (continued).

Metabolite	log2(Fold-Change *NUDT5*^{KO}/parental cell line)	Adjusted P-value (False Discovery Rate)
L-Tryptophan	-0.087426	0.6891
D-Sedoheptulose 7-phosphate	-0.24552	0.69072
L-Histidinol	0.18883	0.69072
Ornithine	-0.11562	0.69227
D-Erythrose 4-phosphate	-0.11024	0.71068
Fumaric acid	0.065943	0.72324
Aminoadipic acid	0.060042	0.76869
Fructose 1,6-bisphosphate	-0.40894	0.76987
dTDP	-0.10804	0.76987
L-2-Hydroxyglutaric acid	-0.086908	0.78958
Guanosine triphosphate	-0.064539	0.78958
Cytidine triphosphate	0.051272	0.78958
Threonine	-0.046978	0.78958
Lauroyl-L carnitine	-0.040914	0.78958
Uric acid	-0.088366	0.79308
Sarcosine	-0.057588	0.81798
FAD	-0.066603	0.83542
Isoleucine	0.090477	0.8388
Cholesterol sulfate	0.057933	0.87432
Serotonin	0.04461	0.87432
1-Methylhistidine	-0.021549	0.87432
ADP	0.021582	0.87738
Glycine	0.029114	0.87754
N-Acetylglutamic acid	-0.028722	0.88096
Stearoyl-L-carnitine	0.025266	0.88096
Taurochenodesoxycholic acid	0.021283	0.88096
Spermine	0.042981	0.89345
Perfluoroheptanoic acid	0.098377	0.91436
Folic acid	-0.05498	0.91436
Thiamine pyrophosphate	-0.065266	0.93085
aminolevulinic acid	0.030479	0.95876
Indole	0.00042574	0.97777
Ursodeoxycholic acid	-0.0067958	0.98147
2-Oxo-4-methylthiobutanoic acid	0.023782	0.98803
L-Valine	0.019802	0.98803
Methionine sulfoxide	-0.0094795	0.98829
Deoxycytidine	0.0040245	0.98829
Tyrosine	0.0074121	0.99603
Phenylalanine	0.01403	0.99822
Coenzyme A	-0.050624	0.99852

Supplemental Table 2: Common non-coding variants associated with 6-mercaptopurine dose intensity in patients with acute lymphoblastic leukemia and eQTL for *NUDT5*.

Variant	Genomic position (hg19)	MAF_{cohort}	Base change	Gene function	Nominal *P* value[a]	Permutation *P* value[b]	Gene expression *P* value[c]	Gene expression score[d]
**rs55713253**	10:12305358	0.0589	C>T	Intergenic (*CD123; CAMK1D*)	0.00186	**0.036**	4.7×10^{-19}	8.9
**rs11257611**	10:12282827	0.0574	C>T	Intronic (*CDC123*)	0.0275	0.268	5.4×10^{-13}	7.2
**rs11818932**	10:12288366	0.0566	G>T	Intronic (*CDC123*)	0.0303	0.292	5.0×10^{-17}	8.4
**rs11818953**	10:12288444	0.0566	G>C	Intronic (*CDC123*)	0.0303	0.292	1.1×10^{-16}	8.3

[a] Nominal *P* value was calculated according to the multiple regression model adjusting for population structure as covariate.

[b] *P* values of the permutation-based analysis to account for multiple testing. A $P < 0.05$ was considered significant.

[c] Corrected *P* values for the association between genotype and gene expression scores were retrieved from the public gene expression database eQTLGen. SNPs were only included if they were located at putative enhancer region according to HaploReg database.

[d] Gene expression scores in blood cells were retrieved from the eQTLGen database.

Abbreviations: eQTL, expression quantitative trait loci; MAF, minor allele frequency.

Supplemental Table 3: Single guide RNA sequences designed for the *NUDIX*-targeted CRISPR/Cas9 screen.

sgRNA identifier	Sequence (5'-3')	sgRNA identifier	Sequence (5'-3')
NUDT1-1	CGCCCATGCTGGTTCCAGC	NUDT12-1	ATCAATTGTCAATAAATCA
NUDT1-2	GTGGCCCGACGACAGCTAC	NUDT12-2	CTTCATTCGTTAGGAACCA
NUDT2-1	ATCCTCTCCTGGTTCCACA	NUDT13-1	ATTTCAGGTTTGTGTAAGG
NUDT2-2	GCCCTGAGGGAGACCCAAG	NUDT13-2	ACTCTTTCAACTCAATGCA
NUDT3-1	TAGTCGCCATCCAGACAGA	NUDT14-1	GAAGAGTTGAATAAGAGAA
NUDT3-2	CTGGAGTAAAAGGGACATT	NUDT14-2	TACAGCTGCTAGGGACCCT
NUDT4-1	TAGAGTTATAACCTTGCAG	NUDT15-1	CAGATGACCTCCAGGGAGT
NUDT4-2	CAGTTATGGTTCACCTTAC	NUDT15-2	CTTTGAGCACATTCTTCCC
NUDT4-3	TTTCACACAAATTCTGTTT	NUDT16-1	CGTGGCCCTGACCCGACGT
NUDT4-4	TAACACTAAGACAGCGAGA	NUDT16-2	TCCTCGAGCGTCAGACGCT
NUDT5-1	AAGTGTTCTCTGCAGCACG	NUDT17-1	CTGCAGCTTGAAGCGAGGA
NUDT5-2	CACTATGAGTGTATCGTTC	NUDT17-2	CCCCCTTTCTGCCCTTTTG
NUDT6-1	AAAATATGTGGAAGTTTCC	NUDT18-1	CTGGCTCCATTCTCCCCGC
NUDT6-2	ATCTTCTTCAGGCTCTGAC	NUDT18-2	GCGCTGCAGCGGGAGGTGA
NUDT7-1	CCGGACGGTGAACAACAAA	NUDT19-1	GAAGCCCTCGGCCGGCGGC
NUDT7-2	TACTCCGTCCTTTTGCCAT	NUDT19-2	TGCAGCGCTCCCCGCACCA
NUDT8-1	CCAGGCGGCAAGTGCGACC	NUDT20-1	AGGGGTACCTAGCAAAATC
NUDT8-2	GTGGTGCACACGGCCCTGC	NUDT20-2	CTTTATTTACTTTTCCTTT
NUDT9-1	TTGAGAGAAAGAGCAAGAA	NUDT21-1	CTGGGTTAAGTTCACCACC
NUDT9-2	CCTGCAGGACGGACTGGAC	NUDT21-2	CTGGTGGTGAACTTAACCC
NUDT10-1	ACACGTACGTTCTGTGCTT	NUDT22-1	GCTCAGGGTGAGCAGCGGC
NUDT10-2	GTCCGAGAGGTGTACGAAG	NUDT22-2	CCCGAAATGAGACCAGTGC
NUDT11-1	CGAGAGGTGTACGAAGAAG		
NUDT11-2	CGAAGAAGCGGGAGTCAAG		

Abbreviations: sgRNA, single guide-RNA; NUDT, NUDIX hydrolase

Supplemental Table 4: List of primers.

Use	Name	5'-3' sequence
CRISPR/Cas9 editing, cloning into pX458	hNUDT5-KO-guide1F	CACCGGAAGTGTTCTCTGCAGCACG
	hNUDT5-KO-guide1R	AAACCGTGCTGCAGAGAACACTTCC
TIDE sequencing	NUDT5-F1	GCCACACGCTGGAAAGTGGT
	NUDT5-R1	CTGGAGGAGCCTCGCTACGA
NUDT5 overexpression	FLAG-NUDT5-F	GATTACAAGGATGACGACGATAAGGAGAGCCAAGAACCAACGGA
	FLAG-NUDT5-R	GGATCCCTAGGAATTTTAAAATTTCAAGAAGGGCA
Cloning into cl20c-IRES-mCherry	NUDT5-EcoRI-F	CTTCTCTAGGCGCCGGGCCACCATGGATTACAAG
	NUDT5-EcoRI-R	GGATCCCTAGGAATTTTAAAATTTCAAGAAGGGCA
Dual-luciferase reporter assay	rs55713253-REF Reference sequence (hg19)	CTGAATTCAAGCAATTCTCCTGCCTTAGCCTCCTGAGTAGCTGGGACCAAAGAGGTACACCACGACATCTGGCTAATTTTTGTATTTTTAGTAGAAATGGGGTTTCACCATATTGGCCAGGCTGGTCTCCAACTCCTGACCTCAAGTGAT**C**AGCCATCTTGGCCTCCCAAAGTGCTGGGGTTACAGGCATAAGCCACTGTGCCCGTACATAATTCTCATTTTAAAAAACTTTTTATTTTTTATTTTTGCAGAACTGTCAAACTCCTTCCTCAAATTTTCTTCCTATTTTATGATTGCTGAC
	rs55713253-ALT Alternative sequence with variant	CTGAATTCAAGCAATTCTCCTGCCTTAGCCTCCTGAGTAGCTGGGACCAAAGAGGTACACCACGACATCTGGCTAATTTTTGTATTTTTAGTAGAAATGGGGTTTCACCATATTGGCCAGGCTGGTCTCCAACTCCTGACCTCAAGTGAT**T**AGCCATCTTGGCCTCCCAAAGTGCTGGGGTTACAGGCATAAGCCACTGTGCCCGTACATAATTCTCATTTTAAAAAACTTTTTATTTTTTATTTTTGCAGAACTGTCAAACTCCTTCCTCAAATTTTCTTCCTATTTTATGATTGCTGAC
	Forward primer	CTAGCAAAATAGGCTGTCCC

Supplemental Figures

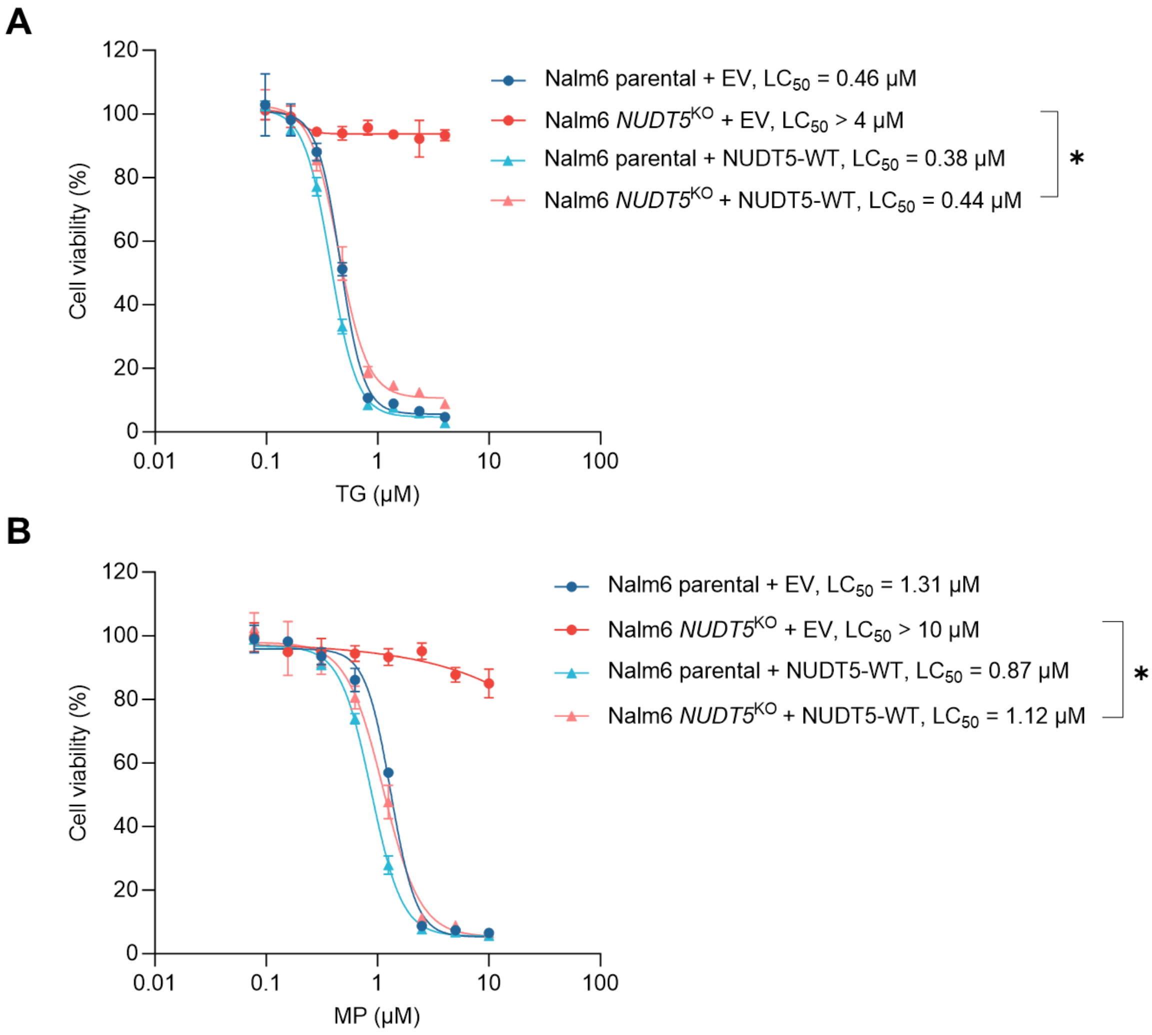

Supplemental Figure 1: The overexpression of *NUDT5* in NUDT5 depleted B-acute lymphoblastic leukemia cells restored thiopurine sensitivity.

Overexpression of *NUDT5* in *NUDT5*^{KO} cells treated with TG (**A**) and MP (**B**). Abbreviations: ALL, acute lymphoblastic leukemia; EV, empty vector; LC_{50}: lethal concentration 50; MP, 6-mercaptopurine; NUDT5, NUDIX hydrolase 5; sgRNA, single guide RNA; TG, 6-thioguanine; WT, wildtype. Data are represented as mean ± standard deviation (n = 3). A two tailed paired t-test was used for the statistical comparisons between groups. * $P < 0.05$; ** P value < 0.01.

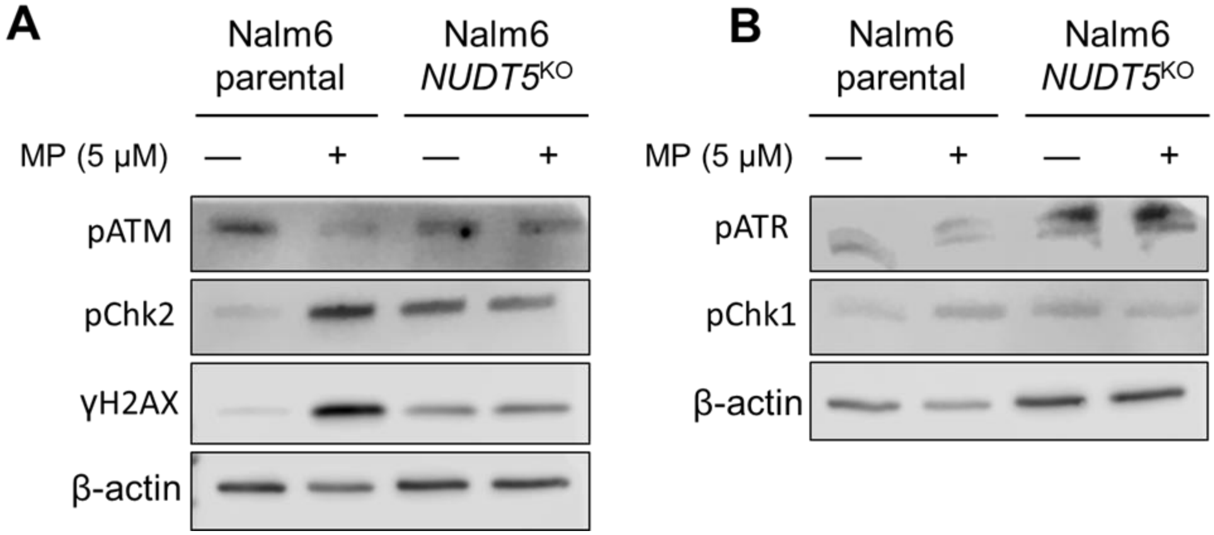

Supplemental Figure 2: NUDT5 depletion abolishes the activation of DNA damage response pathway after treatment with 6-mercaptopurine.

(**A** and **B**) Western blots showing the phosphorylation of DNA double strands breaks sensors ATM/ATR (pATM and pATR, respectively), and activated effectors Chk2, Chk1 and histone 2A (pChk2, pChk1 and γH2AX, respectively), after 24 hours of MP (5 µM) in parental and *NUDT5*^{KO} Nalm6 cells. ß-actin was used as loading control. Abbreviations: γH2AX, phosphorylated histone 2A; MP, 6-mercaptopurine; NUDT5, NUDIX hydrolase 5; pATM/pChk2, phosphorylated ataxia-telangiectasia mutated/checkpoint kinase 2; pATR/pChk1, phosphorylated ataxia telangiectasia and Rad3-related/checkpoint kinase 1.

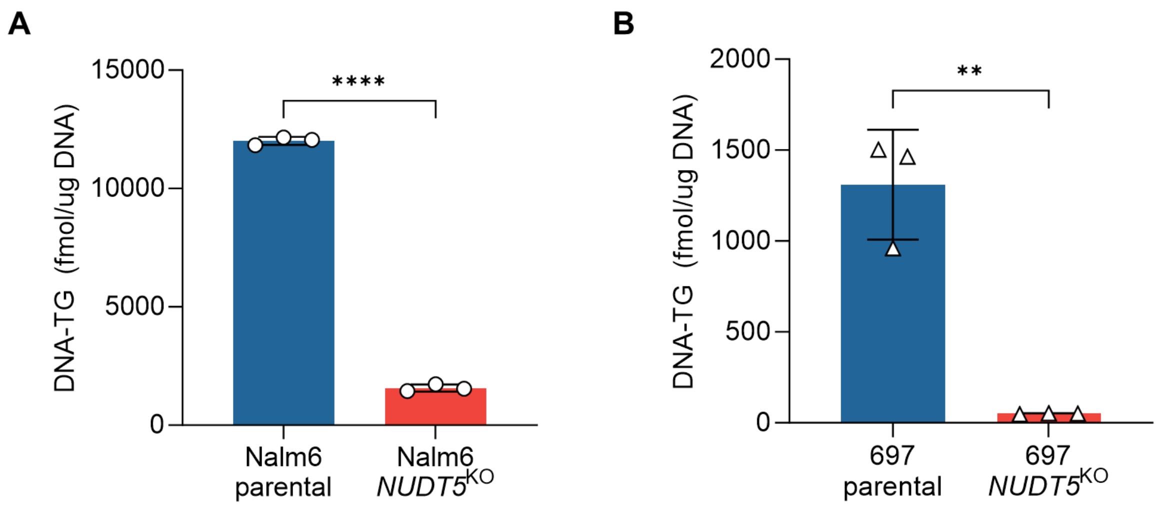

Supplemental Figure 3: NUDT5 disruption alters the incorporation of thiopurine active metabolite into DNA after treatment with 6-thioguanine.

DNA-TG concentrations were measured in parental and *NUDT5*^{KO} Nalm6 (**A**) and 697 (**B**) cells after treatment with 5 µM of TG for 24 hours. Abbreviations: DNA-TG, DNA-thioguanine; NUDT5, NUDIX hydrolase 5; TG, 6-thioguanine. Data are represented as mean ± standard deviation (n = 3). *P* value of two-tailed unpaired t-test. * $P < 0.05$; ** $P < 0.01$.

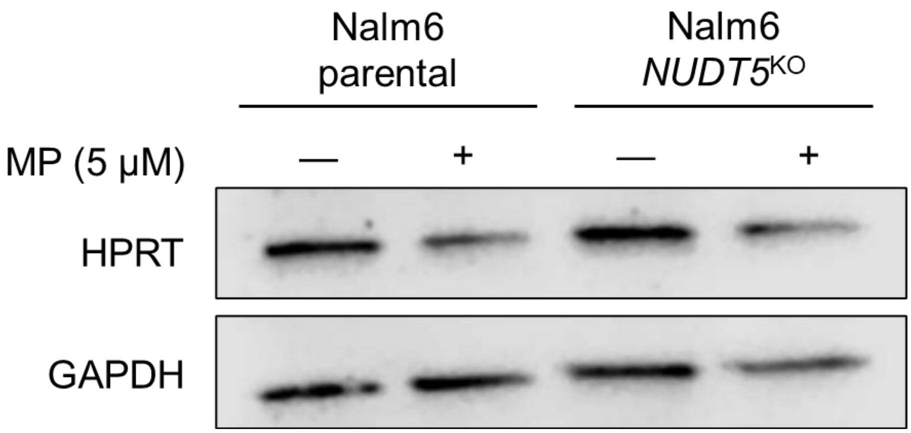

Supplemental Figure 4: *HPRT1* expression in *NUDT5* knockout cells.

The expression of *HPRT1* was verified by western blot in absence and presence of MP for 24 hours. Abbreviations: HPRT, hypoxanthine-guanine phosphoribosyl transferase; GAPDH, glyceraldehyde 3-phosphate dehydrogenase; MP, 6-mercaptopurine; NUDT5, NUDIX hydrolase 5.

References

1. Li W, et al. MAGeCK enables robust identification of essential genes from genome-scale CRISPR/Cas9 knockout screens. *Genome Biol.* 2014;15(12):554.
2. Yang JJ, et al. Inherited *NUDT15* variant is a genetic determinant of mercaptopurine intolerance in children with acute lymphoblastic leukemia. *J Clin Oncol.* 2015;33(11):1235–42.
3. Moriyama T, et al. Comprehensive characterization of pharmacogenetic variants in TPMT and NUDT15 in children with acute lymphoblastic leukemia. *Pharmacogenet Genomics.* 2022;32(2):60-6.
4. Võsa U, et al. Large-scale cis- and trans-eQTL analyses identify thousands of genetic loci and polygenic scores that regulate blood gene expression. *Nat Genet.* 2021;53(9):1300-10.
5. Ward LD, and Kellis M. HaploReg v4: systematic mining of putative causal variants, cell types, regulators and target genes for human complex traits and disease. *Nucleic Acids Res.* 2016;44(D1):D877-81.
