# Supplementary material for: The NUDIX hydrolase NUDT5 regulates thiopurine metabolism and cytotoxicity
Source: J Clin Invest. 2025 Jul 15;135(14):e190443. doi: 10.1172/JCI190443 (PMC12259257; doi:10.1172/JCI190443)
Supplement: Unedited blot and gel images [file jci-135-190443-s073.pdf]

Unedited gels for Figure 2A. Western blot confirming *NUDT5* knockout in the Nalm6 and 697 B-ALL cell lines.

Lanes description:
- L Ladder (EZ Run Prestained Rec Protein Ladder, BP3603500)
- P: Parental cell line
- C1 to C5: *NUDT5*^{KO} clones
- The clone highlighted in **red** was selected for further experiments.

Primary antibody: NUDT5 (EPR7734) Rabbit mAb, Abcam.
Secondary antibody: Anti-rabbit IgG, Amersham ECL HRP-linked Antibody NA9340, Cytiva.

Original

Nalm6 B-ALL cell line

NUDT5 24.3kDa ►

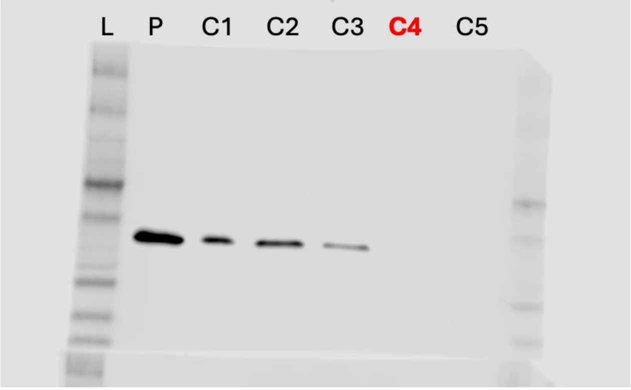

697 B-ALL cell line

NUDT5 24.3kDa ►

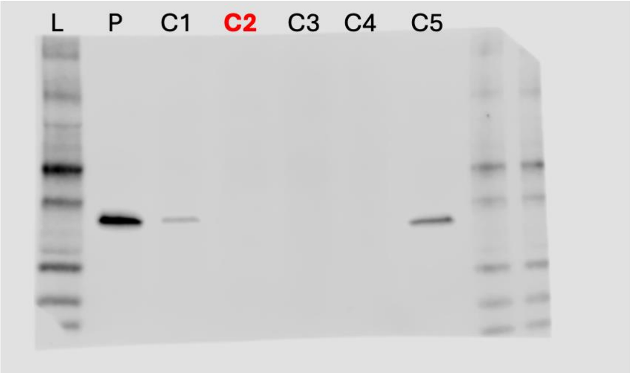

Nalm6 B-ALL cell line

GAPDH 36kDa ►

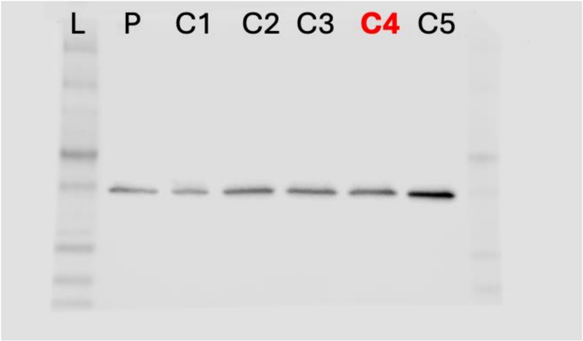

697 B-ALL cell line

GAPDH 36kDa ►

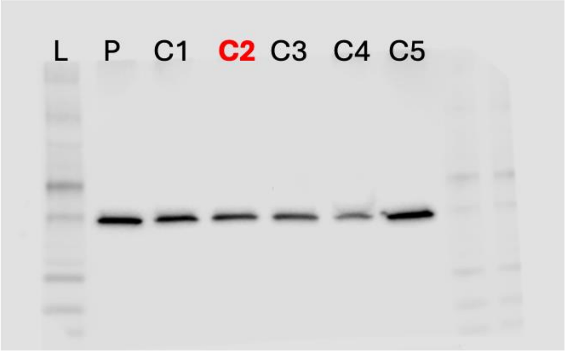

Edited

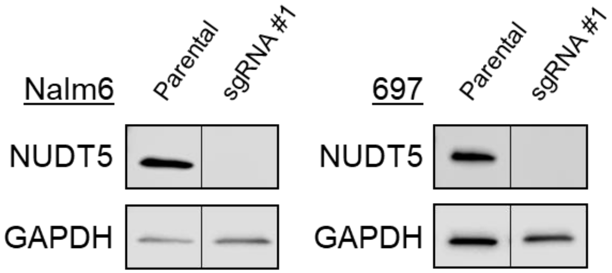

Unedited gels for Supplemental Figure 2. NUDT5 depletion abolishes the activation of DNA damage response pathway after treatment with 6-mercaptopurine.

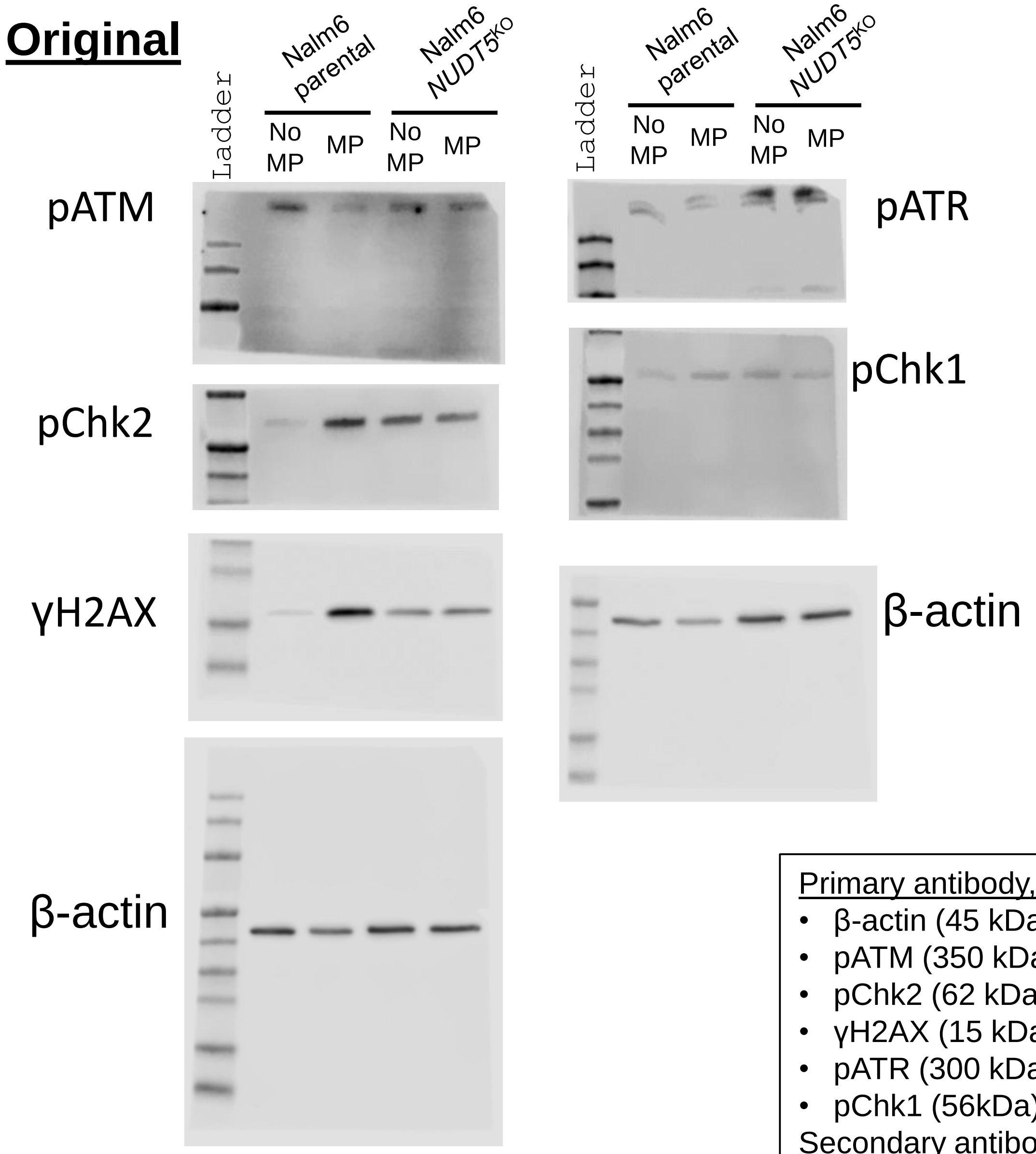

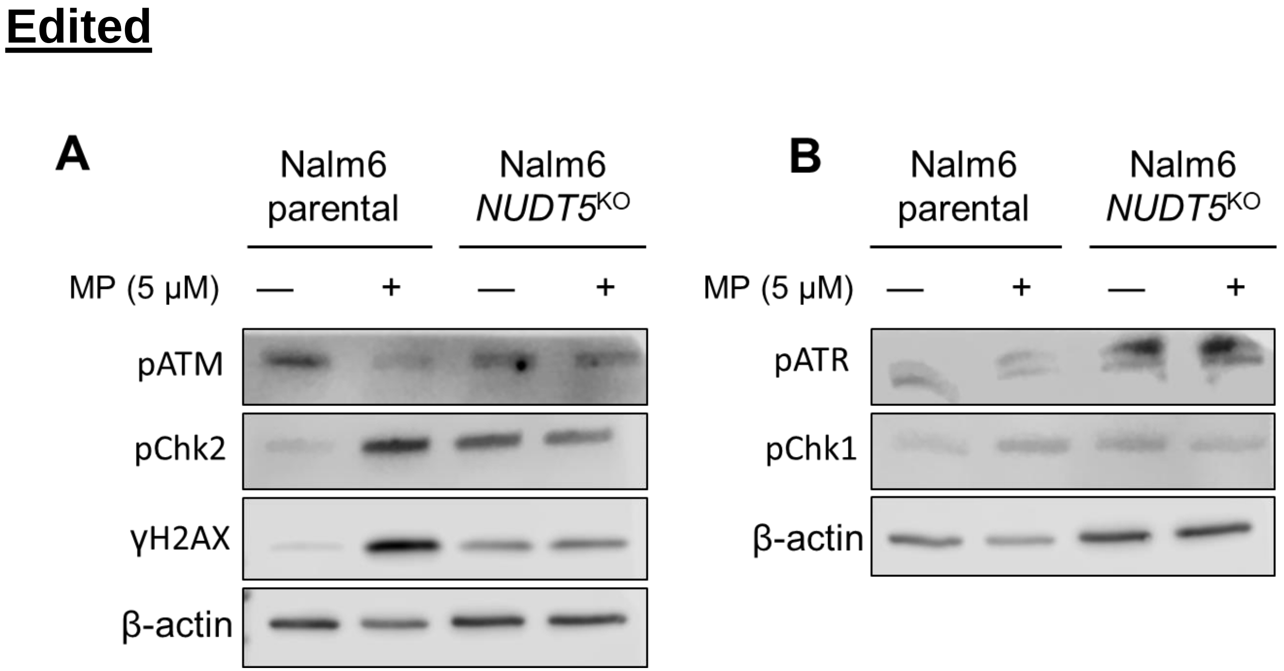

<u>Primary antibody, Ab:</u>

- β-actin (45 kDa), rabbit monoclonal Ab (Cell Signaling Technology, #4967);
- pATM (350 kDa), Ser1981 phosphorylated, rabbit monoclonal Ab (Cell Signaling Technology, #D6H9);
- pChk2 (62 kDa), Thr68 phosphorylated, rabbit monoclonal Ab (Cell Signaling Technology, #E8Q1A);
- γH2AX (15 kDa), Ser139 phosphorylated, rabbit monoclonal Ab (Cell Signaling Technology, #2577);
- pATR (300 kDa), Thr1989 phosphorylated, rabbit monoclonal Ab (Cell Signaling Technology, #D5K8W);
- pChk1 (56kDa), Ser345 phosphorylated, rabbit monoclonal Ab (Cell Signaling Technology, #2348);

<u>Secondary antibody:</u> Anti-rabbit IgG, Amersham ECL HRP-linked Antibody NA9340, Cytiva

<u>Ladder:</u> EZ Run Prestained Rec Protein Ladder, BP3603500

Unedited gel for Supplemental Figure 4. *HPRT1* expression in *NUDT5* knockout cells.

Original

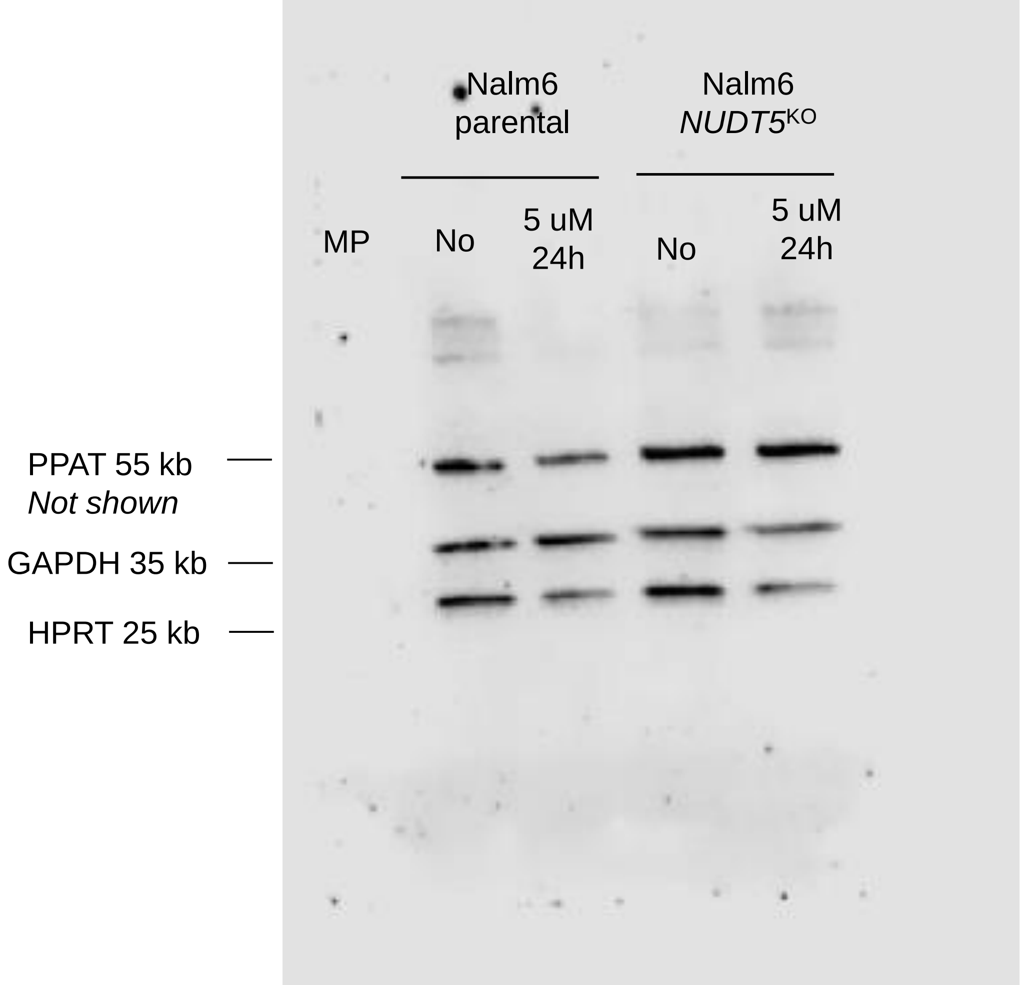

Edited

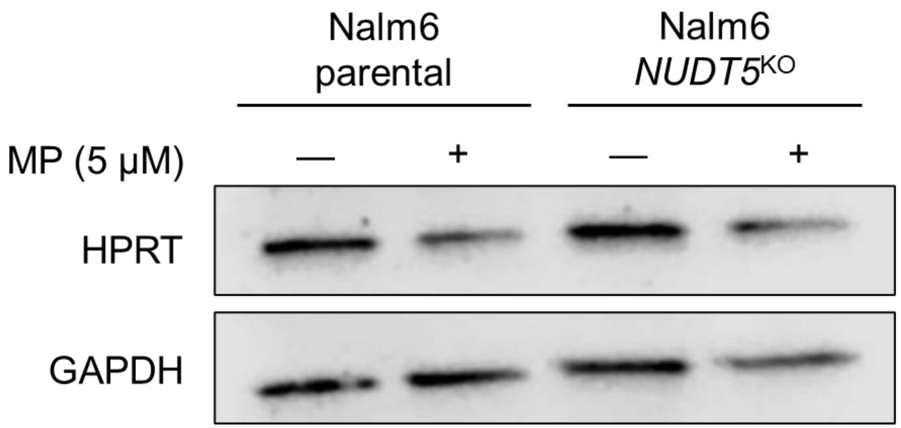

Primary antibodies:

- GAPDH (14C10) Rabbit mAb #2118, Cell Signaling technology;
- HPRT Rabbit mAb #EPR5299, Abcam ;
- PPAT (data not shown in the manuscript), #ab125864, Abcam.

Secondary antibody: Anti-rabbit IgG, Amersham ECL HRP-linked Antibody NA9340, Cytiva
